# Oral nutritional supplementation with dietary counseling improves linear catch-up growth and health outcomes in children with or at risk of undernutrition: a randomized controlled trial

**DOI:** 10.3389/fnut.2024.1341963

**Published:** 2024-07-10

**Authors:** Mandy Y. L. Ow, Nga Thuy Tran, Yatin Berde, Tu Song Nguyen, Van Khanh Tran, Morgan J. Jablonka, Geraldine E. Baggs, Dieu T. T. Huynh

**Affiliations:** ^1^Abbott Nutrition R&D Asia Pacific-Center, Abbott Laboratories, Singapore, Singapore; ^2^Department of Micronutrients, National Institute of Nutrition, Hanoi, Vietnam; ^3^Statistical Services, Cognizant Technologies Solution Pvt. Ltd., Mumbai, India; ^4^Department of General Planning, National Institute of Nutrition, Hanoi, Vietnam; ^5^Abbott Nutrition R&D, Abbott Laboratories, Columbus, OH, United States

**Keywords:** catch-up growth, children, oral nutritional supplements, physical activity, sleep, stunting, undernutrition, underweight

## Abstract

**Introduction:**

Childhood undernutrition is associated with increased morbidity, mortality and a high socio-economic burden.

**Methods:**

**S**upporting **P**ediatric G**R**owth and Health **OUT**comes (SPROUT) is a randomized, controlled trial evaluating the effects of an oral nutritional supplement (ONS) with dietary counseling (DC; *n* = 164) compared to a DC-only group who continued consuming their habitual milk (*n* = 166; NCT05239208). Children aged 24–60 months who were at risk or with undernutrition, as defined by weight-for-age [WAZ] < −1 and height-for-age [HAZ] < −1 according to the WHO Growth Standards, and who also met the criterion of weight-for-height [WHZ] < 0, were enrolled in Vietnam.

**Results:**

ONS + DC had a larger WAZ increase at day 120 (primary endpoint) vs. DC (least squares mean, LSM (SE): 0.30 (0.02) vs. 0.13 (0.02); *p* < 0.001), and larger improvements in all weight, BMI and weight-for-height indices at day 30 and 120 (all *p* < 0.01). Height gain was larger in ONS + DC in all indices, including height-for-age difference [HAD; cm: 0.56 (0.07) vs. 0.10 (0.07); *p* < 0.001], at day 120. ONS + DC had larger arm muscle but not arm fat indices, higher parent-rated appetite, physical activity and energy levels, longer night sleep, fewer and shorter awakenings, and better sleep quality than DC.

**Conclusion:**

Adding ONS to DC, compared to DC-alone, improves growth in weight and height, linear catch-up growth, and health outcomes in children with or at risk of undernutrition.

## 1 Introduction

Childhood undernutrition which includes stunting, wasting, underweight, and micronutrient deficiency or insufficiency is an important public health concern (1). Globally, 149.2 million children under 5 years were stunted, 45.4 million wasted, and 13.6 million severely wasted in 2020 (2). Growth is the primary outcome measure of nutritional status in children (3), and the World Health Organization (WHO) defines stunting as a length- or height-for-age z-score (HAZ) more than two standard deviations (SD) below the WHO Growth Standards median, and wasting as a weight-for-length or weight-for-height z-score (WHZ) more than two SD below the WHO Growth Standards median (4). The highest prevalence of stunting (53%) and wasting (70%) was reported in Asia in 2020 (2). In Vietnam, wasting prevalence has improved from a peak of 11.3% in 2004 to 5.2% in 2019 in children under 5 years. Stunting prevalence has also improved from 43.2% in 2000 but still stands at 19.6% in 2020 (2). Several factors have been found to contribute to childhood undernutrition in Vietnam such as inadequate breastfeeding and poor complementary feeding (5), food insecurity, and poor dietary diversity (6).

Childhood undernutrition is associated with extensive health and socioeconomic burdens, including increased morbidity and mortality (1, 7). The short-term effects include growth impairment and recurrent illness (particularly gastrointestinal and respiratory infections) (1, 8). Long-term effects are far-reaching such as impaired cognitive, psychological, and behavioral development (1, 9); reduced academic performance; shorter adult height (1, 10); and an increased risk for the development of chronic non-communicable diseases (1). Various types of nutritional interventions have been found to be effective in addressing malnutrition in infants and children under 5 years, including the promotion of breastfeeding, provision of complementary foods with education, and supplementary feeding (11, 12). Dietary counseling (DC) is also an integral part of treating undernutrition; however, its effectiveness when administered alone is uncertain (13, 14). Various factors, such as the type of counseling administered, the motivation of the child or parents, individual constraints like time or other stresses inside or outside of the home, or availability of resources or healthy foods, influence the success of DC (15, 16). Because of these limiting factors and the time taken to implement DC advice, adding nutritional supplements alongside DC may be helpful to address undernutrition in a timely manner.

The nutrient composition of nutritional supplements is also important and should address both energy, growth and functional nutrient needs of undernourished children to promote growth, immunity, and development (17). Recommendations for energy and protein requirements for catch-up growth are available from the WHO (18), and recommended nutrient intakes of 30 essential nutrients for children with moderate undernutrition have been proposed by Golden (17). There is also evidence for the potential role of specific nutrients in influencing growth outcomes, such as arginine, vitamin K2, and casein phosphopeptides (CPP). Dietary intake of arginine is associated with increased growth velocity in children (19). Vitamin K2, in the form of menaquinone-7 (MK-7), was shown to be more effective than vitamin K1 in catalyzing the carboxylation of osteocalcin (20). Carboxylated osteocalcin in turn binds to calcium and transports it into the bone, thus facilitating bone mineralization (21). CPPs can chelate key minerals important for growth (e.g., calcium and zinc) and help maintain them in a soluble state (22–24), thus enhancing their absorption in the body, as demonstrated in several preclinical studies.

Polymeric oral nutrition supplements (ONS) that provide a complete blend of macronutrients and micronutrients and have been found to be effective in promoting growth in children aged 9 months to 12 years with undernutrition (25). Providing a complete blend of nutrients is the distinguishing feature of ONS, compared with micronutrient-only supplements, or supplements with one predominant macronutrient type (e.g., lipid-based nutrient supplements). Complete formulas can be effective as poor growth in developing countries has been found to be more common owing to multiple nutrient deficiencies than deficiencies in single micro- or macronutrients (17, 26). In previous randomized controlled trials (RCT) of ONS, improved weight gain has been demonstrated as early as 10 days (27), and consistently at day 30 (27–30). In contrast, the results for height gain are less consistent. One reason may be the inadequate duration of these trials. Among three 90-day RCTs, height gain was larger in the ONS + DC group in only one trial (28) and trended larger but was not significantly different from control in two other RCTs (27, 29). Among longer study durations, a single-arm ONS study found significant increases in height-for-age percentiles (HAP) from 24 weeks onwards (31). A 6-month RCT (32) and another single arm study (33) both showed improved HAZ, compared to control and baseline respectively, at 6-months.

Linear growth has been found to lag behind weight gain by approximately 3 months in undernourished children (34). This was also observed in a single-arm study of ONS, where weight-for-height percentiles increased and plateaued at 4 weeks whereas height-for-age percentiles (HAP) increased gradually, with a statistically significant difference from baseline at 24 weeks (35). Therefore, a longer trial duration of at least 120 days may be better able to detect the effects of an intervention on linear growth. Assessing linear growth in stunting is important as evidence shows that children who recover to a normal height status (HAZ ≥ −1) at 5 years have cognitive function levels similar to children who were never stunted (36). Apart from weight and height indices, body composition assessment was also recently highlighted in an expert consensus paper on growth in infants and young children with faltering growth. The panel described the importance of assessing changes in length-for-age z-score as a simple approximate proxy of lean mass, and also suggested the use of anthropometry-based prediction equations from skinfold thickness measurements for body composition in children from skinfold thickness measurements (37).

We report the findings of a RCT evaluating the efficacy of a new ONS formula with arginine, vitamin K2 and CPP, in combination with DC, compared with DC alone for 120 days, in Vietnamese children aged 24–60 months who were undernourished or at risk of undernutrition. We hypothesize that supplementation with ONS supplying energy, macro- and micronutrients with ingredients to support growth, alongside DC, will improve key nutritional indicators of anthropometric growth and nutrient intake, as well as other health outcomes in undernourished children. These outcomes include arm-anthropometric indices of body composition, functional handgrip strength, and parent-reported levels of the child’s physical activity, appetite, energy, sleep habits, and attentional focusing.

## 2 Materials and methods

### 2.1 Trial design

**S**upporting **P**ediatric G**R**owth and Health **OUT**comes (SPROUT) is a community-based, randomized, open-label, parallel-group, controlled clinical trial of children aged 24–60 months who were at risk of or with undernutrition, involving 1 primary center and 7 preschool satellite sites in Vietnam. Eligible participants were stratified (by gender [male/female] and age [24 to ≤36 months/ > 36 to ≤60 months]) and randomized in a 1:1 ratio to the intervention group (ONS + DC) or control group (DC only). The study duration was 240 days and participants were assessed at baseline, days 30, 120 and 240. Both groups received dietary counseling at the baseline visit, days 30 and 120 and the ONS + DC group additionally received 2 servings of ONS daily for the study duration. This paper reports the study’s primary endpoint of change in weight-for-age z-score (WAZ) from baseline to day 120, and secondary outcomes of anthropometric growth and dietary intake at day 30 and 120. Exploratory outcomes, including bone mineralization and body composition that require a minimum interval of 6 months between baseline and endpoint measurements (38), will be reported in subsequent publication(s) alongside other outcomes assessed at 240 days. The study was conducted according to the guidelines of the Declaration of Helsinki and approved by the Independent Ethics Committee/Institutional Review Board (IRB) of the National Institute of Nutrition in Hanoi, Vietnam (IRB approval code: 1251/VDD-QLKH). The study was prospectively registered at ClinicalTrials.gov (Abbott Nutrition 2022; NCT05239208).

### 2.2 Study setting and population

The study took place in the north of Vietnam at both the main study center (National Institute of Nutrition [NIN]) in Hanoi and 7 preschool satellite sites in Son Duong District, Tuyen Quang Province, Vietnam. After parental consent for screening was obtained, NIN collected anthropometric and demographic information of approximately 3,000 children to screen for children who met the study eligibility criteria. Inclusion criteria were children aged 24–60 months who were undernourished or at risk of undernutrition, as defined by WAZ < −1 and HAZ < −1 according to the 2006 WHO Growth Standards (39), who also met the criterion of weight-for-height-z score [WHZ] < 0; and whose parent(s)/legal guardian (described collectively as “parents” henceforth) were willing to abstain from giving additional non-study ONS. Children were excluded if known to have galactosemia or an allergy or intolerance to any ingredient in the study product, currently drinking products from Abbott Nutrition, had continuous ONS usage for at least 15 days in the past month prior to screening, were born pre-term (birth before 37 weeks of gestation, as reported by a parent), had a birth weight < 2,500 g or > 4,000 g, either parent had a body mass index (BMI) ≥ 27.5 kg/m^2^, had current acute or chronic infections, or diagnosed of clinically significant medical condition (such as severe gastrointestinal, neoplastic, renal, hepatic, cardiovascular, hormonal or metabolic disorders, congenital disease, or genetic disorders) or clinically significant nutritional deficiency requiring specific treatment with another nutritional supplement (other than the study product) in the opinion of the investigator.

### 2.3 Study procedures

Randomization schedules were computer-generated using a biased-coin minimization approach to dynamically assign children to maintain the best possible balance within strata at each site and across sites overall. An electronic data capture system was used to assign subject numbers and allocate participants according to the schedules. As eligible participants were enrolled, they were sequentially assigned a subject number in ascending numerical order within the site and strata combination.

As an open-label study, participants, investigators, and the study team were not blinded to the treatment group allocation. However, as the study product was supplied in plain foil packaging labeled as clinical products for research use, the participants did not know the details of the brand or manufacturer of the ONS provided. Similarly, investigators providing DC were blinded, and clinicians/nurses who performed study outcome assessments were also blinded wherever possible to reduce bias (40).

All study procedures at baseline, days 30, 120 and 240 were completed at the satellite sites (pre-schools). DC was provided to all participants at baseline, day 30 and day 120. DC content was based on local food-based dietary guidelines (41) and the food pyramid (42), including techniques to improve diet quality and help the child to meet daily nutritional requirements according to Vietnamese Recommended Dietary Allowances (RDA) (43). Intervention group participants additionally received two servings of ONS daily for 240 days: administered by teachers at pre-school on weekdays, and by parents at home on weekends or holidays. Baseline assessments and dietary counseling were performed from study day −6 to day 0. Day 1 was the first school day after participants completed all baseline assessments and when the intervention group received their first serving of study product. The ONS (PediaSure^®^; Abbott Laboratories, Vietnam) was packed in a sachet, reconstituted with water up to 225 mL just before consumption. One serving of ONS provides 226 kcal of energy, 6.74 g of protein, 8.81 g of fat, and 29.47 g of carbohydrates. Two servings of ONS daily provide approximately 34 and 54% of recommended energy and protein intake respectively, and more than 50% of each micronutrient recommended intake (Supplementary Table S1). Prior to weekends or holidays, parents met with the product coordinator to receive a sufficient quantity of the study product for consumption during non-pre-school days (public holidays, sick leave, etc.) until the child returned to pre-school.

Owing to the COVID-19 pandemic, some changes to study procedures were made to maintain participant and staff safety while minimizing risks to trial integrity. These included: (1) allowing a wider window for the visit at day 30 (7-day window instead of 5-day) to accommodate the completion of stay-home notices; (2) allowing in-home visits by trained staff to collect anthropometric measurements and deliver study products; and (3) to administer DC or collect study data via telephone, including 24-h recalls and collection of questionnaires.

### 2.4 Concomitant therapy

According to dietary guidelines (44), it is recommended for children over 6 months to consume milk and dairy products appropriate to their age. Therefore, the DC only control group was encouraged to continue with their current milk intake and the ONS + DC intervention group was counseled on how to use the study product as part of their daily diet. Use of any non-study ONS (defined as formulas with an energy density of at least 1 kcal/mL, containing protein, carbohydrate and/or fat, as well as a wide range of micronutrients to supplement or use as the sole source of nutrition), as well as any product from Abbott Nutrition other than study product, was not allowed in either study group.

### 2.5 Outcomes

The primary study endpoint was the change in WAZ from baseline to day 120. Secondary endpoints were changes in anthropometric measures (absolute weight, absolute height, WAZ, weight-for-age percentile [WAP], HAZ, HAP, WHZ, weight-for-height percentile [WHP], BMI-for-age z-score [BMIAZ], BMI-for-age percentile [BMIAP], mid-upper-arm circumference-for-age z-score [MUACAZ] and mid-upper-arm circumference-for-age percentile [MUACAP) based on the WHO Growth Standards) from baseline to day 30 and day 120, energy and macronutrient adequacy and appetite at these time points. Changes in height-for-age difference (HAD) and weight-for-age difference (WAD) from baseline to day 30 and 120 were included in post-hoc analyses. Exploratory endpoints included nutritional status, and improvement in nutritional status from baseline, at days 30 and 120. Nutritional status was defined with anthropometric z-score indices (45): < −3 (severe), ≥ −3 to < −2 (moderate), ≥ −2 to < −1 (mild), and ≥ −1 (normal) for underweight (WAZ), stunting (HAZ) and wasting (WHZ). Other exploratory outcomes included: (a) arm-anthropometric proxy body composition indices (mid-upper-arm muscle circumference (MUAMC), arm muscle area (AMA), arm fat area (AFA), and arm fat index (AFI), (b) lower leg length, (c) muscle strength, (e) attentional focus, (f) sleep, and (g) parent-rated physical activity level, energy and (h) parental satisfaction with child’s and growth and evaluation of improvement from baseline. All endpoints reported in the current paper were assessed at baseline, and days 30, and 120, except for lower leg length, which was assessed only at baseline and day 120.

### 2.6 Outcome assessments

Anthropometric measurements were performed by designated research staff present at pre-schools at each visit. Staff were trained in the techniques of weight, height, MUAC, and triceps skinfold measurements using standardized methods. Weight was measured (in light clothing without footwear) using calibrated electronic weighing scales (Tanita HD 661) and recorded to the nearest 0.1 kg. Standing height was measured (without footwear) using a height stadiometer (ShorrBoard^®^ Infant/Child Measuring Boards) and recorded to the nearest 0.1 cm. MUAC was determined with a flexible, non-stretchable measuring tape and recorded to the nearest 1 mm. Triceps skinfold (TSF) was measured using a skinfold caliper (Harpenden) as an approximation of body fat and recorded to the nearest 0.1 cm. Lower leg length was measured with a knemometer (Shorr Knee-Height Caliper) as the distance from the surface of the right flexed knee to the bottom of the sole, and was performed by the same trained observer without reference to previous recordings.

Sex–age-specific Z-scores and percentiles based on the WHO Child Growth Standards 2006 for weight-for-height, weight-for-age, BMI-for-age, height-for-age, and MUAC-for-age were calculated (39). HAD was calculated as the difference between measured height and the median sex-age-specific height using the same growth standards (46). WAD was calculated using the same concept. HAD is the absolute height deficit relative to the standard, and a reduction in HAD from baseline indicates that a child is growing faster than expected for their age and sex. It is one criterion necessary for demonstrating linear catch-up growth (47). MUAMC was defined as MUAC minus fat mass as measured by TSF thickness (48), using the following formula: $\mathrm{MUAMC}=\mathrm{MUAC}-\left(TSF\times \pi \right)$ . Arm anthropometric measures of MUAC and TSF thickness were used to calculate proxies for body composition indices: AMA, AFA, and AFI using the following equations:

$\mathrm{AMA}=\frac{{\left(\mathrm{MUAC}-TSF\times \pi \right)}^{2}}{4\pi }$ ; (48) AFI=total arm area (TUA)-AMA, where $TUA\;\left(\mathrm{total}\;\mathrm{arm}\;\mathrm{area}\right)=\frac{MUAC^{2}}{4\pi }$ ; (49) $AFI=\frac{AFA}{TUA}\times 100$ (49). AMA is a proxy index of total body muscle mass (48), AFA is an estimate of fat percentage in the upper arm, and AFI is the percentage of the arm that is fat (49).

Dietary intake was assessed with the 24-h dietary recall method using a prompted 24-h recall template by a dietitian or trained researcher to ensure completeness of the dietary record. Nutrient analysis was performed using the Access analysis program with the local food database created by NIN Vietnam. Energy and macronutrient intake and nutrient adequacy were assessed. Nutrient adequacy was defined as meeting 77% of the daily recommended nutrient intake (RNI) (48). Age-specific RNIs from Vietnamese Recommended Dietary Allowances were used.

Muscle strength was assessed using the digital Jamar handgrip strength dynamometer only for older children (36–60 months at baseline). Handgrip strength assessment in younger children has been found to be unreliable and norms are only available for children ≥3 years (50).

Sleep assessments were parent-reported and measured with a questionnaire developed for this study based on existing validated sleep questionnaires for children (51, 52). Parents were asked to report sleep duration (day and night sleep duration), sleep quality on a 10-point visual analog scale (VAS) where 0 and 10 represented “very poor” and “very good” overall sleep quality, respectively, and the number of times and duration of night sleep awakenings.

Attentional focusing was assessed with the attentional focusing subscale of the Early Childhood Behavior Questionnaire (ECBQ) (53) or the Children’s Behavior Questionnaire (CBQ) (54) for children aged 24–36 months and > 36 months, respectively, at baseline. The same questionnaire was administered across visits for each child, even if the child progressed to the next age band at subsequent visits. Scores from the ECBQ and CBQ were reported separately rather than combined, as recommended by the questionnaire developer.

Child physical activity and appetite levels over a 24-h period were rated on a 10-point VAS. Parental satisfaction with child growth was reported using a 10-point VAS and included questions on satisfaction with the child’s weight, height, growth, muscle, bone, teeth, skin, hair, nails, and eyes.

### 2.7 Adverse events

Adverse events (AEs) were collected by a standard method where all untoward medical occurrences temporally associated with the study, whether or not related to the study product, were recorded. AEs were reported by parents and caregivers and recorded by the study sites. Both non-serious and serious AEs were medically confirmed and assessed for causality by the study physicians. AEs were coded and grouped according to the Medical Dictionary for Regulatory Activities (MedDRA version 21.1) terminology.

### 2.8 Statistical analysis

Sample size was calculated based on the primary outcome, and the between-group difference in the mean change of WAZ from baseline to 120 days. A sample size of 132 in each group was calculated to have 80% power to detect a between-group difference of at least 0.08 in the mean changes in WAZ from baseline to 120 days, assuming a common standard deviation of 0.231 (30) using a two-group t-test with a 0.05 two-sided significance level. Target enrolment was 165 per group assuming a 20% attrition. The nQuery software (version 8) was used for sample size calculations (nQuery, 2017).

All statistical analyses were performed on the intention-to-treat (ITT) and per-protocol (PP) populations using SAS^®^ Version 9.4 software (SAS Institute Inc). The ITT population included all randomized participants who received at least one study feeding (in the intervention group), regardless of the actual feeding consumed. The PP population included only participants who consumed at least 75% of prescribed ONS intake (ONS + DC group only), did not consume medications or supplements that could affect the primary or secondary outcomes for more than 25% of the intervention period, did not consume any non-study ONS or Abbott Nutrition product, and met all study eligibility criteria.

Continuous baseline demographic variables were summarized with means and SD, whereas categorical variables were summarized as number of participants (n) and as percentages (%). Primary, secondary and post-hoc anthropometric endpoints were analyzed with ANCOVA, with changes from baseline to day 30 or 120 as the dependent variables. Main effects were site, treatment and gender, with treatment × gender as an interaction effect. Baseline values of the outcome variable and age were included as covariates of the ANCOVA models. To explore whether treatment effects were different across nutritional status subgroups, additional ANCOVA analyses including interaction terms of treatment × nutritional status were performed for WAZ, HAZ, WHZ, BMIAZ, and MUACZ indices. Nutritional status subgroups were defined as < −2 vs. ≥ −2 for WAZ and HAZ, and < −1 vs. ≥ −1 for WHZ, BMIAZ, and MUACZ endpoints. Different cut-offs were used because the inclusion criteria in this study was < − 1 for WAZ and HAZ, and < 0 for WHZ, and cut-offs were chosen to achieve balanced numbers of participants in each nutritional status subgroup. Nutritional status categories at baseline were compared between groups using chi-squared tests and any improvement in nutritional status from baseline analyzed using generalized estimating equation (GEE) with main effects for site, treatment, gender, visit, interaction effects of treatment × visit and treatment × gender, with age and baseline nutritional status as covariates. Subgroup analyses were also performed for improvement in nutritional status based on baseline severity: (a) WAZ, HAZ, or WHZ ≥ −2 at baseline; and (b) WAZ, HAZ or WHZ < −2 at baseline.

Other continuous endpoints including energy and macronutrient intake, arm anthropometric indices, 10-point VAS scores, sleep, and sleep awakening durations, and CBQ and ECBQ scores were analyzed with repeated measure ANCOVA with main effects for site, treatment, gender, visit, interaction effect of treatment × visit, treatment × gender, and with age and baseline values of the corresponding outcome as covariates. Residuals from parametric analysis were utilized to check for deviation from normality by a combination of methods (stem-and-leaf plot of residuals, normality plot, Shapiro–Wilk test).

Categorical outcome variables, namely nutrient adequacy against Vietnam estimated average requirements were analyzed with chi-square test. Lower leg length at 120 days was analyzed using two-sample two-sided Wilcoxon rank sum test. Number of night awakenings was analyzed using GEE with main effects for site, treatment, gender, visit, interaction effects of treatment × visit and treatment × gender, and with age and baseline values of the outcome as covariates. To analyze the effect of product intake compliance on growth, multiple linear regression analyses including age (in months), gender, site, baseline values of the outcome and the number of servings consumed in the 120-day intervention period as predictors were performed with each primary and secondary anthropometric endpoint as the dependent variable. All tests of hypotheses were two-sided, 0.05 level tests, except tests for interaction effects which were two-sided 0.10 level tests.

## 3 Results

### 3.1 Participants

**Figure 1:**
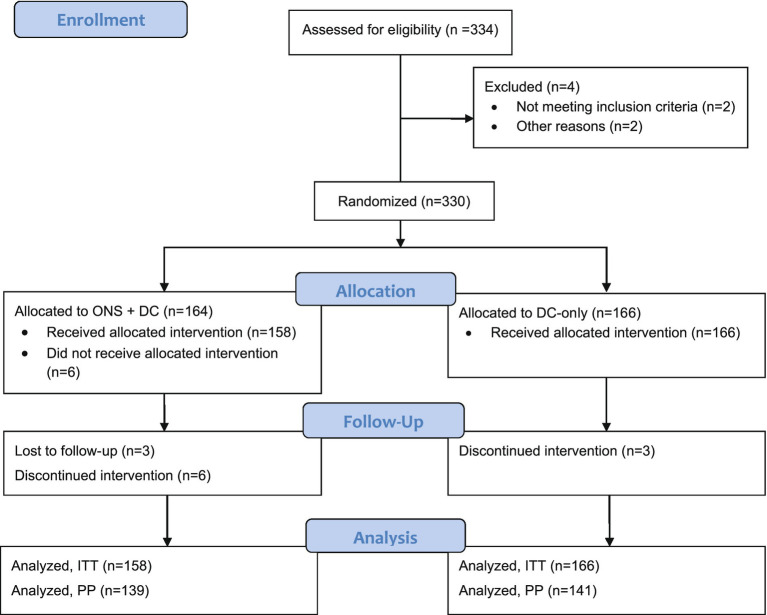
CONSORT study flow diagram. DC, dietary counseling; ITT, intention-to-treat; ONS, oral nutritional supplements; PP, per-protocol.

Initially, 332 children were screened for eligibility, of whom 2 did not meet the inclusion criteria. A total of 330 eligible children were randomized in this study (164 intervention, 166 control) as shown in the CONSORT flow diagram (Figure 1) (55). Six intervention participants who did not receive any ONS (due to parents’ change in mind on study participation or that the child could not accept the taste of the study product) were excluded from the ITT population. In total, 324 and 280 participants were included in the ITT and PP populations, respectively. Results of ITT and PP analyses were similar and only ITT results are presented subsequently. The study was conducted from January 2022 to December 2022 including the intervention period. Children were enrolled in 2 batches: the first batch of 282 (85%) children were enrolled between 14 and 21 January 2022, and the second batch between 13 and 15 April 2022. In between these enrolment waves, there was an escalation of the COVID-19 situation in Vietnam and a COVID-19 lockdown affecting the study sites was enforced between 8 February–4 April 2022. During this period, pre-schools were closed, and people were encouraged to work from home and stay at home whenever possible with safe-distancing measures enforced.

**Figure 2:**
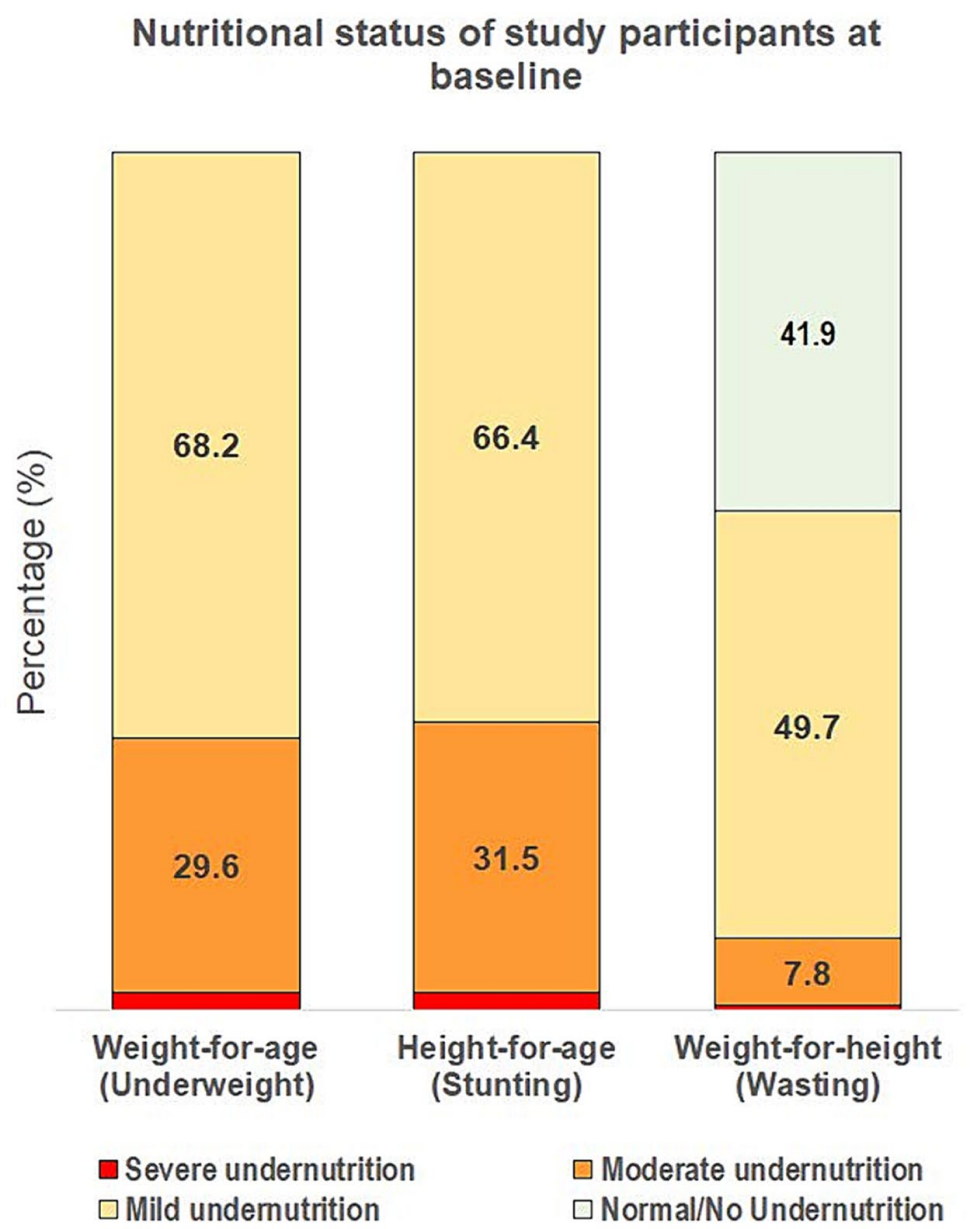
Severity of underweight, stunting, and wasting of the overall study population at baseline. Severity of undernutrition is defined by z-score cut-offs as: ≥ −1 for normal/no undernutrition; < −1 to ≥ −2 for mild; < −2 to ≥ −3 for moderate; and < −3 for severe undernutrition, using weight-for-age, height-for-age, and weight-for-height indices for underweight, stunting and wasting, respectively.

Within the ITT population, 88.6% of children were in the older age strata (37 and 287 children aged ≥24–36 and > 36–60 months, respectively) and the mean overall age was 46.5 months (standard error [SE]: 0.5) at enrolment, with approximately equal males (48.8%) and females (Table 1). At baseline, all children were at least mildly stunted and mildly underweight. Additionally, 58.1% of children were also at least mildly wasted, whereas 41.9% had a normal weight-for-height status with WHZ between <0 and ≥ −1 (Figure 2). Most children were mildly underweight (68.2%), and the majority were also mildly stunted (66.4%), defined as WAZ or HAZ < −1, respectively. Fewer children were moderately underweight (29.6%) or moderately stunted (31.5%), and very few were severely underweight or stunted (Figure 2). 49.7% of all children had mild wasting and very few children in the study population were moderately or severely wasted (7.8 and 0.6%, respectively). Key baseline characteristics are reported in Table 1 and additional characteristics in Supplementary Table S2.

**Table 1:** Key baseline sociodemographic and anthropometric characteristics of participants and households.

	ONS + DC (*n* = 158)	DC only (*n* = 166)
Child characteristics		
Age (months)	46.8 ± 0.7	46.3 ± 0.6
Age categories, n (%)		
> 24 to ≤36 months	19 (11.4)	18 (11.4)
> 36 to ≤60 months	140 (88.6)	147 (88.6)
Gender, n (%)		
Male	76 (48.1)	81 (48.8)
Female	82 (51.9)	85 (51.2)
Anthropometry		
Weight-for-age (z-score)	−1.91 ± 0.05	−1.85 ± 0.04
Height-for-age (z-score)	−1.84 ± 0.04	−1.86 ± 0.04
Weight-for-height (z-score)	−1.23 ± 0.05	−1.12 ± 0.05
BMI-for-age (z-score)	−1.07 ± 0.05	−0.96 ± 0.05
MUAC-for-age (z-score)	−1.23 ± 0.05	−1.19 ± 0.05
Gestational age (weeks)	38.5 ± 0.1	38.5 ± 0.1
Exclusive breastfeeding (months)	5.14 ± 0.13	5.33 ± 0.13
Total breastfeeding^a^ (months)	8.82 ± 0.37	8.74 ± 0.37
Parental and household characteristics		
Mother’s highest level of education, n (%)		
Postgraduate degree or doctorate	0 (0.0)	0 (0.0)
College or university degree	11 (7.0)	9 (5.5)
Associate or technical degree	15 (9.6)	7 (4.2)
High school diploma	69 (43.9)	88 (53.3)
Secondary school	50 (31.8)	46 (27.9)
Primary school or less	12 (7.6)	15 (9.1)
Annual income quintiles, n (%)		
1st Quintile	40 (25.3)	43 (26.2)
2nd Quintile	28 (17.7)	38 (23.2)
3rd Quintile	38 (24.1)	33 (20.1)
4th Quintile	31 (19.6)	29 (17.7)
5th Quintile	21 (13.3)	21 (12.8)
Number of children up to 13 years old in home	2.16 ± 0.06	1.99 ± 0.06

Data are presented as mean ± SE for continuous variables and n (%) for categorical variables. ^a^Include partial and exclusive breastfeeding duration. BMI, body mass index; DC, dietary counseling; LSM, least squares mean; MUAC, mid-upper arm circumference; ONS, oral nutritional supplement; SE, standard error.

### 3.2 Primary outcome, weight and weight indices

**Figure 3:**
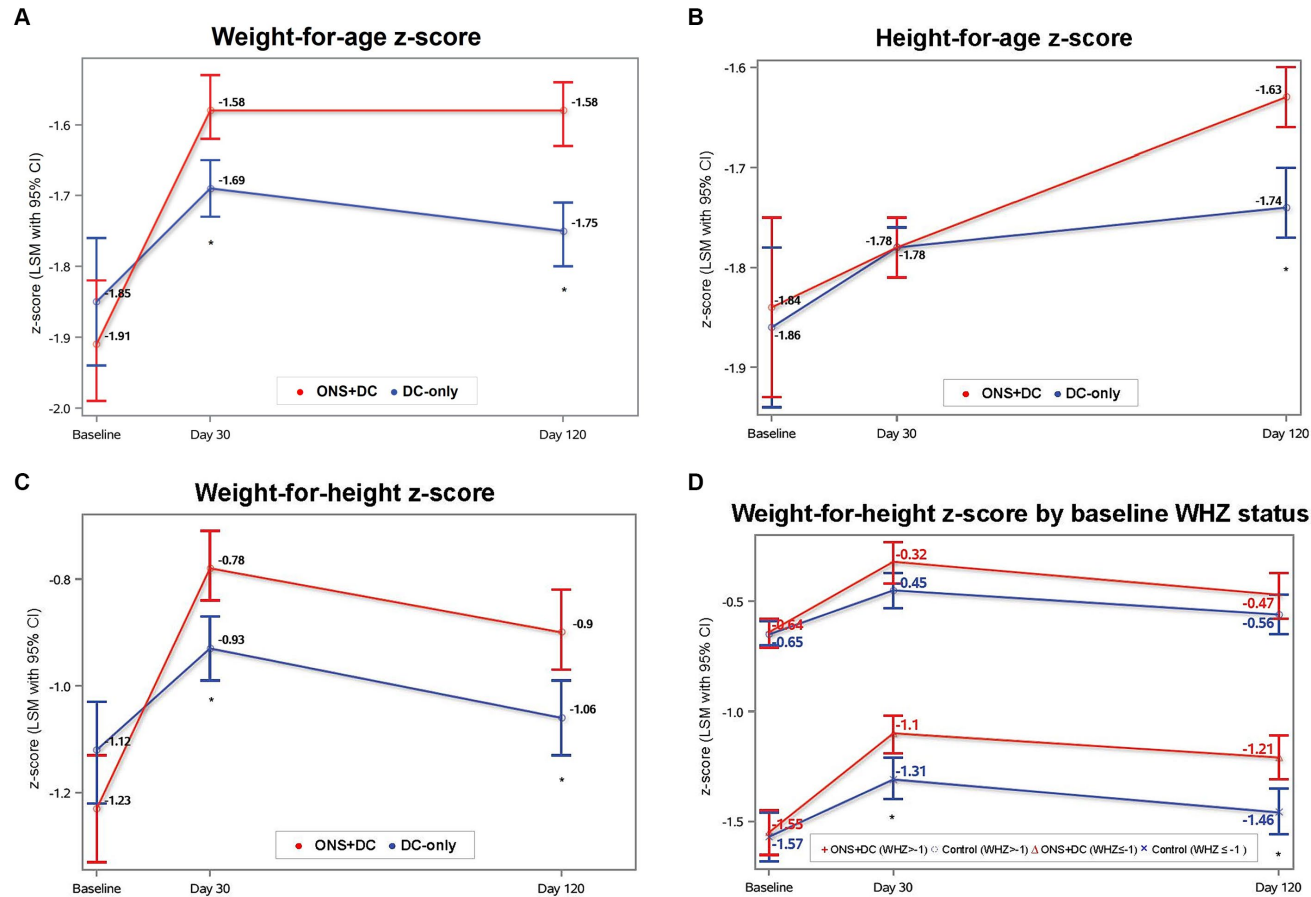
Anthropometric indices at baseline, day 30, and day 120 by treatment group for **(A)** WAZ, **(B)** HAZ, **(C)** WHZ, and **(D)** WHZ by baseline WHZ status. Baseline values were ANOVA LSM ± SE and day 30 and 120 values were from repeated-measures ANCOVA estimates. *indicates *p* < 0.05 for between-group comparisons with ANCOVA for that timepoint. CI, confidence interval; HAZ, height-for-age-z score; LSM, least squares mean; SE, standard error; WAZ, weight-for-age z score; WHZ, weight-for-height-z score.

The ONS + DC group had greater increases in weight, WAZ, WAP and WAD at both day 30 and 120 than the DC-only control group (*p* < 0.001 for all parameters and at both timepoints; Table 2). The primary endpoint of this trial was achieved in that the ONS + DC group had a larger WAZ gain than the control group at day 120 (WAZ change from baseline to day 120, least squares mean [LSM] ± SE: 0.30 ± 0.02 vs. 0.13 ± 0.02 [control]; mean difference: 0.17 ± 0.03; *p* < 0.001; Table 2; Figure 3A). Mean WAZ and WAP increased at day 30 from baseline in both groups and remained stable to day 120 in the ONS + DC group, whereas there was a slight decline in the control group at day 120 (Table 2; Figure 3A).

**Table 2:** Effect of ONS intervention on anthropometric parameters from baseline to days 30 and 120.*

	Visits	ONS + DC	DC Only	Difference	*p*-value
Weight (kg)	Baseline	12.51 ± 0.07	12.59 ± 0.07	−0.08 ± 0.09	–
	Change at Day 30	0.64 ± 0.03	0.44 ± 0.03	0.21 ± 0.05	**<0.001**
	Change at Day 120	1.04 ± 0.04	0.73 ± 0.04	0.31 ± 0.05	**<0.001**
WAZ	Baseline	−1.91 ± 0.05	−1.85 ± 0.04	−0.06 ± 0.06	–
	Change at Day 30	0.30 ± 0.02	0.18 ± 0.02	0.12 ± 0.03	**<0.001**
	Change at Day 120	0.30 ± 0.02	0.13 ± 0.02	0.17 ± 0.03	**<0.001**
WAP	Baseline	4.39 ± 0.32	4.91 ± 0.32	−0.53 ± 0.43	–
	Change at Day 30	3.38 ± 0.27	1.84 ± 0.26	1.54 ± 0.36	**<0.001**
	Change at Day 120	3.55 ± 0.3	1.49 ± 0.29	2.06 ± 0.40	**<0.001**
WAD	Baseline	− 3.43 ± 0.07	−3.35 ± 0.07	−0.08 ± 0.09	–
	Change at Day 30	0.43 ± 0.03	0.22 ± 0.03	0.20 ± 0.05	**<0.001**
	Change at Day 120	0.32 ± 0.04	0.01 ± 0.0.04	0.31 ± 0.05	**<0.001**
Height (cm)	Baseline	94.28 ± 0.18	94.17 ± 0.18	0.11 ± 0.25	–
	Change at Day 30	0.87 ± 0.06	0.85 ± 0.06	0.02 ± 0.08	0.833
	Change at Day 120	2.98 ± 0.07	2.52 ± 0.07	0.46 ± 0.09	**<0.001**
HAZ	Baseline	−1.84 ± 0.04	−1.86 ± 0.04	0.02 ± 0.06	–
	Change at Day 30	0.05 ± 0.01	0.05 ± 0.01	0.00 ± 0.02	0.876
	Change at Day 120	0.20 ± 0.02	0.09 ± 0.02	0.11 ± 0.02	**<0.001**
HAP	Baseline	4.89 ± 0.34	4.79 ± 0.33	0.10 ± 0.45	–
	Change at Day 30	0.75 ± 0.22	0.72 ± 0.21	0.03 ± 0.29	0.929
	Change at Day 120	2.66 ± 0.26	1.11 ± 0.26	1.54 ± 0.35	**<0.001**
HAD	Baseline	−7.65 ± 0.18	−7.76 ± 0.18	0.11 ± 0.24	–
	Change at Day 30	0.12 ± 0.06	0.11 ± 0.06	0.01 ± 0.08	0.843
	Change at Day 120	0.56 ± 0.07	0.10 ± 0.07	0.46 ± 0.09	**<0.001**
WHZ	Baseline	−1.23 ± 0.05	−1.12 ± 0.05	−0.10 ± 0.07	–
	Change at Day 30	0.40 ± 0.03	0.24 ± 0.03	0.16 ± 0.04	**<0.001**
	Change at Day 120	0.31 ± 0.04	0.15 ± 0.04	0.16 ± 0.05	**0.002**
WHP	Baseline	14.50 ± 0.93	16.50 ± 0.92	−2.01 ± 1.25	–
	Change at Day 30	10.16 ± 0.81	5.82 ± 0.8	4.34 ± 1.09	**<0.001**
	Change at Day 120	7.68 ± 0.88	4.08 ± 0.85	3.60 ± 1.16	**0.002**
BMIAZ	Baseline	−1.07 ± 0.05	−0.96 ± 0.05	−0.11 ± 0.07	–
	Change at Day 120	0.38 ± 0.03	0.23 ± 0.03	0.16 ± 0.04	**<0.001**
	Change at Day 120	0.24 ± 0.04	0.10 ± 0.04	0.14 ± 0.05	**0.006**
BMIAP	Baseline	17.87 ± 1.07	20.20 ± 1.05	−2.33 ± 1.43	–
	Change at Day 30	10.70 ± 0.87	5.98 ± 0.85	4.72 ± 1.17	**<0.001**
	Change at Day 120	6.59 ± 0.92	3.02 ± 0.9	3.58 ± 1.24	**0.004**
MUACZ	Baseline	−1.23 ± 0.05	−1.19 ± 0.05	−0.04 ± 0.07	–
	Change at Day 30	0.05 ± 0.03	−0.06 ± 0.03	0.11 ± 0.04	**0.006**
	Change at Day 120	0.06 ± 0.04	−0.04 ± 0.03	0.10 ± 0.05	**0.030**
MUACP	Baseline	14.20 ± 1	15.33 ± 0.98	−1.13 ± 1.34	–
	Change at Day 30	0.89 ± 0.57	−0.97 ± 0.57	1.86 ± 0.77	**0.016**
	Change at Day 120	1.30 ± 0.76	−0.46 ± 0.73	1.76 ± 1.00	0.078

Data are presented as LSM ± SE. Change values: ANCOVA. *p*-values in bold are *p* < 0.05, underlined *p*-values are *p* < 0.10 and ≥ 0.05. ANCOVA, analysis of covariance; BMI, body mass index; BMIAP, BMI-for-age percentile; BMIAZ, BMI-for-age z-score; DC, dietary counseling; HAD, height-for-age difference; HAP, height-for-age percentile; HAZ, height-for-age z-score; LSM, least squares mean; MUACP, mid-upper arm circumference percentile; MUACZ, mid-upper arm circumference z-score; ONS, oral nutritional supplement; SE, standard error; WAD, weight-for-age difference; WAP, weight-for-age percentile; WAZ, weight-for-age z-score; WHP, weight-for-height percentile; WHZ, weight-for-height z-score.

### 3.3 Height, height indices, and proportionate weight indices

Height gain in the ONS + DC group was also greater in all measures (absolute height, HAZ, HAP and HAD) than the control group at day 120 (*p* < 0.01 for all measures) but not on day 30 (Table 2). At day 120, the ONS + DC group had a larger absolute height gain of 2.98 in cm (SE: 0.07) compared with 2.52 cm (SE: 0.07) in the DC group (*p* < 0.001). Using relative height-for-age indices, both groups increased HAP and HAZ from baseline (Table 2; Figure 3B), with the ONS + DC group showing twice the growth improvements at day 120 (HAZ: 0.20 ± 0.02 vs. 0.09 ± 0.02 [control]; HAP: 2.66 ± 0.26 vs. 1.11 ± 0.26 [control]; both *p* < 0.001; Supplementary Table S3). Using absolute height deficits, the DC group had a minimal change of 0.1 cm (SE: 0.07) in HAD from baseline whereas the ONS + DC group had a 0.56 cm (SE: 0.07) improvement in HAD (*p* < 0.001) at day 120 (Table 2). 77.4% in the ONS + DC group, compared to 59.3% in the DC group, had a reduction in absolute height deficit, indicated with a positive change in HAD (odds ratio (95% CI): 2.42 (1.46, 4.00); *p* = 0.001).

Proportionate weight, assessed with weight-for-height (Figure 3C), BMI-for-age and MUAC z-scores and percentiles, were also higher in the ONS + DC group at both day 30 and 120 than the control group (*p* < 0.05 for all measures and timepoints, except for MUAC percentiles gain at day 120: *p* = 0.078; Table 2).

### 3.4 Anthropometric growth by baseline nutritional status

In exploratory analyses to investigate whether treatment effects were different by baseline nutritional status (WAZ, HAZ: ≥ −2 vs. < −2; WHZ: ≥ −1 vs. < −1), the treatment-by-baseline-nutritional-status interaction term was significant only for WHZ at day 120 (interaction term: *p* = 0.074) but not for WAZ or HAZ at any time point. At day 120, WHZ gain was larger in the ONS + DC group compared with the control group only in the subgroup with wasting at baseline (WHZ < −1 subgroup; WHZ gain: 0.40 ± 0.05 vs. 0.15 ± 0.05 [control], *p* < 0.001). In children without wasting at baseline, there was no difference in WHZ gain between groups (WHZ ≥ −1 subgroup; WHZ gain: 0.19 ± 0.06 vs. 0.13 ± 0.05 [control], *p* = 0.45; Figure 3D).

### 3.5 Nutritional status at days 30 and 120

At baseline, nutritional status was not different between groups (Supplementary Table S2). At day 120, stunting status was different between groups (*p* = 0.015), with 3 times as many children in the ONS + DC group (11.6%) having recovered to a normal height status (HAZ ≥ − 1) compared to the DC group (3.7%; Table 3). ONS + DC had also higher odds of less severe stunting at day 120, and less severe underweight and wasting at both days 30 and 120 (Table 3). Similarly, in subgroup analyses of mild undernutrition at baseline, more children in ONS + DC recovered to a normal height status (16.2% [ONS + DC] vs. 5.8% [DC]; *p* = 0.016) and normal weight-for-height status (34.5% [ONS + DC] vs. 19.4% [DC]; *p* = 0.036) at day 120, whereas the difference did not reach statistical significance for recovery to normal weight status (18.1% [ONS + DC] vs. 10.1% [DC]; *p* = 0.092; Supplementary Table S4A). In subgroups with moderate-to-severe undernutrition at baseline, i.e., subgroups meeting WHO’s definition of undernutrition (HAZ, WAZ or WHZ < −2), 32.7% vs. 19.0% recovered from moderate stunting (*p* = 0.104), 36.0% vs. 19.2% recovered from moderate underweight (*p* = 0.058) and 66.7% vs. 33.3% (*p* = 0.085) recovered from moderate wasting in the ONS + DC and DC groups, respectively (Supplementary Table S4B). These differences did not reach statistical significance likely due to smaller sample sizes (total *n* = 107, 102 and 27, respectively, for HAZ, WAZ or WHZ < − 2 respectively, at baseline).

**Table 3:** Nutritional status at day 30 and day 120.

	Undernutrition Severity (n, %)			Odds ratio of lower severity in ONS + DC (95% CI)^†^	*p*-value^†^
	ONS + DC	DC only	*p*-value*		
**Day 30**					
Stunting					
Normal	5 (3.2)	7 (4.3)	0.839	–	0.218
Mild	107 (67.7)	107 (65.6)			
Moderate–Severe	46 (29.1)	49 (30.1)			
Underweight					
Normal	17 (10.8)	12 (7.4)	0.569	1.97 (1.06, 3.66)	**0.030**
Mild	102 (64.6)	109 (66.9)			
Moderate–Severe	39 (24.7)	42 (25.8)			
Wasting					
Normal	100 (63.3)	90 (55.2)	0.256	**#**	**#**
Mild	50 (31.6)	66 (40.5)			
Moderate–Severe	8 (5.1)	7 (4.3)			
**Day 120**					
Stunting					
Normal	18 (11.6)	6 (3.7)	**0.015**	4.45 (1.79, 11.05)	**0.003**
Mild	103 (66.5)	108 (66.7)			
Moderate–Severe	34 (21.9)	48 (29.6)			
Underweight					
Normal	19 (12.3)	11 (6.8)	0.096	4.13 (2.15, 7.94)	**<0.001**
Mild	102 (65.8)	102 (63.0)			
Moderate–Severe	34 (21.9)	49 (30.2)			
Wasting					
Normal	76 (49.0)	75 (46.3)	0.766	1.72 (1.17, 2.54)	**0.006** ^**#**^
Mild	57 (36.8)	66 (40.7)			
Moderate–Severe	22 (14.2)	21 (13.0)			

*Chi-square test. ^**†**^Odds ratio of ONS + DC to DC-only group having a lower severity at day 30 or 120: GEE (site, treatment, gender, treatment*gender, visit, treatment*visit, with age (in months) and baseline value as covariates). #Overall treatment effect *p*-value across day 30 and day 120 (treatment-by-visit interaction not significant) across Day 30 and 120. GEE, generalized estimating equation.

### 3.6 Energy and macronutrient intake and adequacy

At baseline, the majority of the study population had inadequate energy, carbohydrate, and fat intake (33.1, 33.7 and 16.1% adequacy for energy, carbohydrate and fat, respectively). Regular milk consumption was allowed, and DC group reported consuming cow’s milk, fortified beverages, growing up milk, and other beverages such as plant-based milk. A higher proportion of the ONS + DC group achieved nutrient adequacy at days 30 and 120 for energy, carbohydrate, and fat adequacy (*p* < 0.05, for all comparisons). At day 120, ≥ 80% of the ONS + DC group achieved adequacy for energy, protein and carbohydrate intake and almost 70% achieved fat intake adequacy (Table 4). In contrast, only 45.1, 51.9, and 23.5% achieved energy, carbohydrate, and fat adequacy, respectively, in the DC group. Protein adequacy was high (88.2%) at baseline in the overall study population. At day 30, no difference in protein adequacy between groups was observed. However, at day 120, 100% of the ONS + DC group achieved protein adequacy, a higher rate than the control (100% vs. 93.2% [control]; *p* < 0.001). Mean intakes of energy and all macronutrients were also higher in the ONS + DC group at days 30 and 120 (*p* < 0.001, for all comparisons; Table 4). The increase in mean energy and macronutrient intake at day 120 from baseline, expressed as a percentage of baseline intake, in the ONS + DC group was 51% vs. 9% (DC) for energy, 45% vs. 7% (DC) in for protein, 41% vs. 11% (DC) for carbohydrate, and 82% vs. 9% (DC) in for fat intake.

**Table 4:** Energy and macronutrient intake and adequacy at baseline, day 30, and day 120.*

		Intake			Achieving nutrient adequacy^^^ (%)		
	Follow-up visits	ONS + DC	DC Only	*p-*value	ONS + DC	DC Only	*p*-value
Energy (kcal)	Baseline	835.0 ± 17.9	867.60 ± 17.4	–	49 (31.0)	58 (35.2)	–
	Day 30	1207.0 ± 20.9	957.40 ± 20.6	**<0.001**	132 (83.5)	80 (49.1)	**<0.001**
	Day 120	1264.5 ± 19.0	949.97 ± 18.7	**<0.001**	145 (92.9)	73 (45.1)	**<0.001**
Protein (g)	Baseline	33.0 ± 1.0	34.69 ± 1.0	–	136 (86.1)	149 (90.3)	–
	Day 30	45.0 ± 1.0	37.18 ± 1.0	**<0.001**	157 (99.4)	157 (96.3)	0.062
	Day 120	47.9 ± 0.9	36.99 ± 0.9	**<0.001**	156 (100.0)	151 (93.2)	**<0.001**
Carbohydrate (g)	Baseline	128.0 ± 2.7	130.15 ± 2.7	–	48 (30.4)	61 (37.0)	–
	Day 30	175.7 ± 3.3	144.40 ± 3.2	**<0.001**	129 (81.6)	83 (50.9)	**<0.001**
	Day 120	180.8 ± 3.0	144.05 ± 3.0	**<0.001**	135 (86.5)	84 (51.9)	**<0.001**
Fat (g)	Baseline	21.2 ± 0.9	23.00 ± 0.8	–	24 (15.2)	28 (17.0)	–
	Day 30	35.8 ± 1.0	25.52 ± 1.0	**<0.001**	87 (55.1)	48 (29.4)	**<0.001**
	Day 120	38.6 ± 0.9	25.01 ± 0.9	**<0.001**	108 (69.2)	38 (23.5)	**<0.001**

*LSM ± SE. ^**^**^Based on age- and sex-specific estimated average requirement, derived from Vietnamese RNIs. Day 30/120 analyses: repeated-measures ANCOVA. p-values in bold are *p* < 0.05. Underlined *p*-values are *p* < 0.1 and > 0.05. ANCOVA, analysis of covariance; DC, dietary counseling; GEE, generalized estimating equation; LSM, least square mean; ONS, oral nutritional supplement; SE, standard error.

### 3.7 Lower leg length, arm anthropometry, and handgrip strength

No difference was observed in lower leg length between the groups at day 120. The ONS + DC group had larger arm muscle indices: a larger MUAMC and a larger AMA at post-baseline visits than the control group (MUAMC [cm] LSM ± SE: 12.42 ± 0.03 vs. 12.32 ± 0.03 [control], *p* = 0.027; AMA [cm^2^], LSM ± SE: 12.31 ± 0.06 vs. 12.12 ± 0.06 [control], *p* = 0.026). No differences between groups were found in the arm fat indices of AFA or AFI, or in dominant or non-dominant handgrip strength (for children ≥36 months at enrolment only) at post-baseline visits.

### 3.8 Sleep and attentional focusing

Parents of participants in the ONS + DC group reported improved child sleep in four out of the five domains evaluated in the sleep questionnaire: a longer night sleep duration (hours, LSM ± SE: 9.41 ± 0.05 vs. 9.26 ± 0.05 [control], *p* = 0.015), better sleep quality (10-point VAS score: 8.81 ± 0.06 vs. 8.39 ± 0.06 [control], *p* < 0.001), shorter duration of night awakenings (minutes of awakening: 0.83 ± 0.27 vs. 1.99 ± 0.27 [control], *p* = 0.001), and fewer night awakenings (odds of ONS + DC group having fewer awakenings: 1.62 [95% CI, 1.05–2.49]; *p* = 0.029). Duration of day sleep was not significantly different between groups. Most children (88.6%) completed the CBQ attentional focusing subscale as they were 36–60 months old at enrolment. In this age subgroup, participants in the ONS + DC group had better attentional focusing scores than participants in the control group (mean score, LSM ± SE: 4.70 ± 0.05 vs. 4.55 ± 0.05 [control], *p* = 0.047). There was no significant difference in attentional focusing scores among the younger age group completing the ECBQ (*n* = 37; Supplementary Table S5).

### 3.9 Parent-rated appetite, physical activity, energy levels

Parent-rated appetite, physical activity, and energy/activity levels, as indicated on a 10-point VAS, were higher in the ONS + DC group than DC over days 30 and 120 (appetite score LSM ± SE: 7.10 ± 0.09 vs. 6.50 ± 0.08 [DC], *p* < 0.001; physical activity score LSM ± SE: 8.21 ± 0.07 vs. 7.90 ± 0.06 [DC], *p* < 0.001; energy/activity score LSM ± SE: 8.15 ± 0.07 vs. 7.94 ± 0.07 [control], *p* = 0.021; Supplementary Table S5).

### 3.10 Parental satisfaction and evaluation of improvement in child’s growth and health

At baseline, there were no differences in parental satisfaction of child’s weight, height, growth, muscle, and bone health on 10-point VAS between groups. Parental satisfaction scores in all domains were higher in ONS + DC group post-baseline (mean scores: 6.97–7.50; Supplementary Table S6). When asked to evaluate improvements in their child’s health and growth from baseline, ratings were also higher in the ONS + DC group in all domains including growth, muscle, bone, teeth, alertness/curiosity/eagerness, skin, nails, hair, and eyes (all *p* < 0.001; Supplementary Table S7).

### 3.11 Product intake compliance and association with weight, height, and proportionate weight

Overall, ONS intake compliance in the intervention group was high (median compliance at day 30: 98.6%, interquartile range [IQR]: 97.1–100%; median compliance at day 120: 95.9%, IQR: 92.3–98.3%). The number of servings consumed in the 120-day period was positively associated with height and HAZ gain at day 120 (per 225 mL serving, height gain [cm] beta: 0.0056, *p* = 0.018; HAZ gain beta: 0.0013, *p* = 0.0194) while inversely associated with WHP gain at day 120 (per 225 mL serving, WHP gain beta: −0.087, *p* = 0.0248). However, after adjusting for height gain, the number of servings consumed was no longer significantly associated with WHP gain (per 225 mL serving, WHP gain beta: −0.065, *p* = 0.094), suggesting that the negative association with WHP was partly attributed to the height gain of the children. The number of servings of ONS consumed over the 120-day period was not significantly associated with any other weight, height, or proportionate weight measures or indices in these multiple linear regression analyses.

### 3.12 Adverse events

AEs were mild for the majority of participants with no significant difference in severity between the study groups. A total of 53.8% of participants in the ONS + DC group and 60.6% in the control DC-only group reported at least one AE. The proportion of participants with any AE, serious or non-serious, was not significantly different between the study groups (*p* = 0.24). AEs commonly associated with gastrointestinal tolerance (i.e., constipation, diarrhea, and vomiting) were reported in 3% of participants with no differences observed between the ONS + DC and DC-only groups. The majority of AEs (98.1%) were non-serious with the remaining 1.9% of participants (6 participants) reporting a serious AE (SAE). This included 1 participant from the ONS + DC group and 5 participants from the control DC-only group. None of the SAEs were related to the study interventions. Overall, the study ONS product (i.e., ONS + DC) was found to be safe and well-tolerated.

## 4 Discussion

The current study demonstrates that supplementation with ONS alongside DC for 120 days significantly improved key nutritional indicators, namely improved growth in weight, height, linear catch-up growth, and increased energy and macronutrient intake and adequacy in children aged 24–60 months at risk of or with undernutrition, compared with DC alone encouraging habitual milk consumption. This study also found several other benefits from the added ONS strategy, in terms of larger arm muscle but not fat indices, better parent-reported child’s sleep habits, child’s appetite, physical activity and energy levels, as well as higher attentional focusing scores in the ONS + DC group.

Compared with the DC-only group, the ONS + DC group improved growth in weight and height consistently across absolute and the various age-and-sex-standardized z-score or percentile indices. As expected, intervention effects on linear growth were detected later, as weight, weight-for-height, BMI- and MUAC-for-age gains were greater in the ONS + DC group from day 30, whereas height gains were greater only at day 120. Interaction analyses by baseline nutritional status suggest that intervention effects on stunting and underweight status were not different between mild and moderate-to-severe subgroups at baseline. For weight-for-height, ONS + DC only increased WHZ if at least mild wasting was present at baseline. Analyses of nutritional status categories showed that three times as many children in the ONS + DC group recovered to a normal height-for-age status at day 120 compared to the DC-only group.

Using absolute height deficits, the DC group showed minimal change in HAD whereas the ONS + DC group had a mean HAD improvement of 0.56 cm at day 120. A positive change in HAD has been interpreted as a faster-than-normal growth velocity compared to children of their age and sex (36, 46, 56, 57), which fulfills one criterion of catch-up growth (46, 47). Although a wide variety of ‘catch-up growth’ definitions have been used earlier (37, 58), Frongillo et al. have recently specified the criteria necessary to demonstrate catch-up growth (47). These criteria are: an initially reduced growth velocity, the removal of an inhibiting condition, and a subsequent higher-than-normal growth velocity (47).

We did not measure growth velocity prior to enrolment in our study. However, since study inclusion criteria were HAZ < −1 at enrollment, and normal weight (> 2,500 g and < 4,000 g) at birth, enrolled children likely had preceding periods(s) of reduced growth velocity prior to enrollment. DC alone partly corrected the inhibitory condition of inadequate nutrient intake at baseline, as indicated by the slightly improved nutrient adequacy rates at follow-up visits in the DC group. Nevertheless, 50% had still not met energy and carbohydrate adequacy, and 70% had not met fat adequacy at day 120. In contrast, the ONS + DC group had only 7.0, 14.0, and 30.0% of children not meeting energy, carbohydrate, and fat adequacy, respectively. Taken together, study findings indicate that adding ONS to DC to improve nutrient intake and to close the adequacy gap is an effective strategy in promoting linear catch-up growth.

To the best of our knowledge, very few nutritional intervention studies have reported effects on HAD. An earlier study of multiple micronutrient powder supplementation in 6–23 months old Ethiopian children found that though HAD decline was less in the intervention group relative to the control group, the intervention group still had a worsening height deficit from baseline to 1 year (mean HAD of −1.11 cm (baseline) and − 4.2 cm (at 1 year)), demonstrating that the intervention had impact on linear growth relative to the control group but no evidence of catch-up growth with respect to the growth standards (59). Another study found that provision of quality maize for 1 year did not improve HAD or HAZ compared to the control group, and all groups had increased height deficits after 1 year (60). Our study finding of 0.56 cm HAD improvement in the ONS + DC group in 120 days is encouraging, particularly as children in this study were at least 24 months old at baseline (mean age: 46 months), supporting previous analyses which show that linear catch-up growth can occur beyond 24 months or after the first 1,000 days of life (61).

Several observations suggest that the ONS + DC group displayed appropriate growth, rather than accelerated growth (or excessive weight gain), which might have adverse effects on long-term health, such as later life obesity (37). First, none of the children in this study exceeded WHZ > 2 at day 120, the WHO definition of overweight in children. Second, weight-for-age and weight-for-height indices increased from baseline to day 30 and remained stable or declined slightly, respectively, relative to day 120 (Figures 3A,C), whereas height-for-age indices increased continuously to day 120. Third, interaction analyses indicated that only children with wasting at baseline (WHZ < −1) in the ONS + DC group had larger WHZ gains than the control group. Among children without wasting at baseline (42% of the study population), there was no difference in WHZ gain between groups (Figure 3D). The greater WHZ gain observed in the subgroup with wasting is expected, as previous studies demonstrate that weight gain in terms of weight-for-height correction occurs before linear catch-up growth can occur, during the nutritional rehabilitation of concurrently stunted and wasted children (34, 62, 63). On the other hand, children with stunting but not wasting require only weight gain that is proportionate to their height gain. This requires an adequate intake of growth nutrients (such as zinc, sulfur, phosphorous, vitamins D, C, K and copper) to support bone and lean tissue synthesis for linear growth, without excess weight gain (63). The lack of difference between treatment groups in WHZ in the subgroup without wasting indicate that ONS + DC group did not put on excess weight relative to the height gained in 120 days. Finally, proxy body-composition indices with arm anthropometry also showed that arm muscle indices, but not arm fat indices, were larger in the ONS + DC group compared with the control group. Together, these observations do not suggest continuous and disproportionate weight gain but show improvements in height and body composition assessments (37).

Current findings have some similarities to previous RCTs comparing ONS (with or without DC) to DC or placebo control groups, in similar populations of undernourished children without underlying chronic medical conditions. Results on weight gain are generally consistent across studies, with larger weight gain observed in the ONS groups by day 30 and up to day 90 (27–30) or at 6 months (32). On the other hand, effects on height gain are mixed. Greater height gain in the ONS group was reported by Alarcon et al. in 36–60 months old children in Philippines and Taiwan (28), and by Lebenthal et al. in 3–9 years old children in Israel but only among the compliant ONS subgroup (32). In contrast, two RCTs in India of children aged 24–72 months and 24–48 months found trends but no significant differences between groups (27, 29), and a RCT in China of children aged 30–60 months by Sheng et al. (2014) also found no difference between groups (30). These inconsistent findings could be due in part to differences in study populations, including age and baseline stunting severity. In the current study and Lebenthal et al., baseline stunting was slightly more severe (mean HAZ of −1.84 and − 2.04, respectively) compared to studies that did not find an ONS effect on linear growth [baseline mean HAZ in these studies: −1.66 (29), − 1.4 (27), and − 0.6 (30)]. Nevertheless, Alarcon et al. included children with milder or comparable baseline stunting [baseline HAP: 19.2 (z-score approximate: −0.88)] but also demonstrated improved height gain in the ONS group from day 60. Notably, Alarcon et al. used the highest ONS dosage (providing an average of 540 kcal per day) among these six ONS studies. In contrast, the two other 90-day studies which prescribed lower dosages (between 224 and 448 kcal per day) found no differences between groups at day 90 (27, 29). Both the current study and Lebenthal et al. also found positive correlations between ONS consumption volume and HAZ gain, supporting that ONS dosage may be an important factor in height gain. Other intervention-related factors, such as duration and the specific ONS formulation might also contribute to these mixed findings. Lebenthal et al. prescribed a relatively lower ONS dose (providing 354 kcal per day) but for a longer duration of 6 months and found better height gain in the compliant ONS subgroup. Both Sheng et al. and the current study used a 120-day intervention period (providing 400 kcal and 450 kcal per day, respectively), with only the latter showing that the ONS group had improved height gain. As these studies utilized different ONS formulations, the specific ONS composition and nutrients included to support growth, such as arginine and CPP that were included in the current study formula, may also contribute to varying effects of ONS on linear growth.

Physical activity is an important child health outcome as it mediates behavioral and brain development (64). Undernutrition is associated with decreased physical activity, which in turn decreases a child’s exploration of the environment and interaction with caregivers, adversely affecting behavioral and brain development (64). In our study, VAS ratings of both the child’s physical activity and energy (worded as “child’s energy/activity level, as indicated by being alert, curious and eager to explore and learn”) improved, indicating that physical activity and energy, including exploratory interest, increased in the ONS + DC group. Previous trials of nutritional supplementation in undernourished children have shown mixed results in terms of improving physical activity and exploratory behavior (65–67). In this study, the provision of both energy and a complete blend of macro- and micronutrients including iron and zinc might have contributed to the increased activity and energy levels observed (67). Iron and zinc have also been found to be involved in appetite regulation, and their supplementation has resulted in improved appetite in young children with evidence of growth faltering in previous studies (27, 31, 68). The prevalence of zinc deficiency in Vietnam is high—estimated to be 51.9 and 12.9% for zinc- and iron-deficiencies, respectively, among children aged 6–75 months in 2009 (69); and 41.4 and 2.4%, respectively, among children aged 6–9 years in 2016 (70). In undernourished children, such as in the current study population, prevalence of deficiencies is likely to be higher. 2 servings of ONS in this study contributed to 60 and 110% of daily zinc and iron RNI respectively, thus could explain the increase in appetite, physical activity and energy ratings of the ONS + DC group.

Prior research on sleep and nutritional status in young children has focused primarily on the associations of shorter sleep duration with higher risks of overweight and obesity (71, 72). Sleep duration and linear growth has been studied less frequently and with mixed results. Longer sleep duration in infants and children <2 years have been found to predict or precede greater linear growth in two studies (73, 74) but not in another study (75). In cross-sectional studies, anemia and stunting were associated with reduced sleep duration and increased night awakening, and serum ferritin levels and anemia were associated with different sleep patterns in children (76). To the best of our knowledge, this is the first study to investigate the association between correction of undernutrition and benefits to sleep duration and quality. Interestingly, these results show that the duration of night awakening, length of night sleep, and overall sleep quality were improved with fewer night awakenings during the study period in the ONS + DC group. This might be due to: (1) provision of micronutrients in ONS including Vitamin D, iron, magnesium, and zinc (77–80), which are associated with improved sleep duration and sleep quality; (2) increased intake of protein, specifically tryptophan, which is known to play a key role in sleep regulation (81); and (3) the increase in physical activity, which is associated with improved sleep quality (82). Sleep is a highly dynamic developmental process that evolves rapidly during the first few years of life and plays a key role in cognition and physical growth, which reciprocally impact one another (83), and thus should be further investigated in future research.

Undernutrition in early life also has a significant adverse impact on cognitive neurodevelopment (84). Encouragingly, evidence shows that among stunted children, cognitive and neurodevelopmental deficits can potentially be recovered before 8 years, particularly in those whose nutritional status has improved (84). In our study, the scores for attentional focusing were improved for participants in the ONS + DC group. Two meta-analyses have identified benefits for ONS on cognition in children at risk of undernutrition in low and middle-income countries. Notably, ONS containing multiple micronutrients were found to have the largest positive effect on cognition in both studies (26, 85). As multiple nutritional deficiency or insufficiency is relatively common in developing countries, it was suggested that the provision of multiple nutrients is more likely to bridge the gap and prepare the optimal foundation for rapid brain development in early childhood (26).

Finally, in our study, AEs reported were typical in nature for the population, such as respiratory infection, fever, cough and runny nose. The majority of AEs were mild (93%) and deemed not related or probably not related to the study product (97%), with no significant differences in incidence between the ONS + DC and DC-only groups. AEs commonly associated with gastrointestinal tolerance (i.e., constipation, diarrhea, and vomiting) were infrequently reported and not significantly different between the study groups. Overall, these findings support the safety and tolerance of the study ONS intervention (i.e., ONS + DC). The rates of GI tolerance AEs reported in the current study were comparable in type and incidence to those found in other studies investigating ONS in undernourished children (86, 87).

### 4.1 Study strengths and limitations

Several strengths are of note. The study design was strong as a randomized study with a control group provided with usual DC care. Although an open-label study, investigators providing DC were blinded, clinicians/nurses who performed study outcome assessments were also blinded wherever possible, and ONS identity or details was not disclosed to participants to reduce study bias. The control group was allowed to continue consuming habitual milk, including cow’s milk, fortified beverages or formula milk as long as the formula did not meet the definition of ONS, defined in this study as a formula providing 1 kcal of energy per mL and providing a wide range of macro- and micronutrients. The 120 days duration to assess linear growth, and the inclusion of body composition indices provided opportunities to assess quality growth beyond weight gain. This study also had a high rate of compliance and a low number of participants lost to follow-up. Child health outcomes that have been less frequently studied were also included in this study, such as sleep, attentional focusing, and physical activity, providing important new information on the potential benefits of correcting child undernutrition (88).

This study has limitations. First, the study population was heterogeneous in terms of undernutrition severity (ranging from mild to severe) and forms of undernutrition. In terms of severity, our study enrolled fewer children in the moderate–severe subgroups, particularly with very few children who were moderately or severely wasted. Our study hence has limited generalizability to these children. Despite the heterogeneity in severity, interaction analyses do not suggest that there is a difference in treatment effects between mild and moderate–severe stunting and underweight subgroups. In terms of heterogeneity in undernutrition form, 41.9% of children were stunted and underweight but not wasted, and 58.1% had all three forms of undernutrition. We found significant interaction effects, revealing that the ONS + DC increased WHZ more than DC-only group only in the subgroup with baseline wasting. Second, we used parent-reported tools to assess several exploratory outcomes, including physical activity level, appetite level, energy, sleep behavior and the ECBQ/CBQ for attentional focusing. Parent-reported outcomes can be more prone to recall bias, particularly in an open-labeled trial, compared with performance-based or objective assessments of these outcomes. Nevertheless, these are exploratory outcomes and it is possible that the improvement of growth and other health outcomes reflects the improvement of overall health followed the correction of undernutrition. These promising findings encourage further research using gold-standard and/or objective assessment methods, such as laboratory polysomnography for sleep research, performance-based cognitive testing, and objective measurements using actigraphy and activity trackers for sleep and physical activity assessment.

## 5 Conclusion

Our study showed consistent improvement of all anthropometric indices with the ONS + DC strategy, indicating that ONS added to DC promotes weight gain by day 30 and linear catch-up growth at day 120 when compared to DC alone encouraging habitual milk intake. The addition of ONS to DC enhanced nutrient intake and appropriate catch-up growth, and current findings indicate that correcting undernutrition with ONS may improve other important child outcomes such as physical activity, energy, sleep habits, appetite, and attentional focusing which should be confirmed in future research.

## Data availability statement

The datasets presented in this article are not readily available because ethical restrictions imposed by the IRB prevents public sharing of the data for this study in children. The data used in this publication is owned by Abbott Nutrition. Data access request will be evaluated by Abbott Nutrition in consideration of IRB requirements. Interested researchers will need to sign a research collaboration agreement with Abbott. Requests to access the datasets should be directed to mandyyenling.ow@abbott.com.

## Ethics statement

The studies involving humans were approved by Independent Ethics Committee/Institutional Review Board (IRB) of the National Institute of Nutrition in Hanoi, Vietnam. The studies were conducted in accordance with the local legislation and institutional requirements. Written informed consent for participation in this study was provided by the participants’ legal guardians/next of kin.

## Author contributions

MO: Data curation, Formal analysis, Methodology, Visualization, Writing – original draft, Writing – review & editing. NT: Data curation, Investigation, Project administration, Supervision, Writing – review & editing. YB: Data curation, Formal analysis, Visualization, Writing – review & editing. TN: Investigation, Project administration, Writing – review & editing. VT: Investigation, Project administration, Writing – review & editing. MJ: Data curation, Methodology, Writing – review & editing. GB: Formal analysis, Methodology, Supervision, Writing – review & editing, Data curation. DH: Conceptualization, Formal analysis, Methodology, Project administration, Supervision, Writing – review & editing.

## References

[1] Black RE Allen LH Bhutta ZA Caulfield LE de Onis M Ezzati M. Maternal and child undernutrition study group. Maternal and child undernutrition: global and regional exposures and health consequences. Lancet. (2008) 371:243–60. doi: 10.1016/S0140-6736(07)61690-0 18207566

[2] United Nations Children's Fund, World Health Organization, World Bank Group. Levels and trends in child malnutrition: UNICEF/WHO/the World Bank Group joint child malnutrition estimates: Key findings of the 2021 edition. Geneva: World Health Organization (2021).

[3] Becker PJ Carney LN Corkins MR Monczka J Smith E Smith SE. Consensus statement of the academy of nutrition and dietetics/American Society for Parenteral and Enteral Nutrition: indicators recommended for the identification and documentation of pediatric malnutrition (undernutrition). J Acad Nutr Diet. (2014) 114:1988–2000. doi: 10.1016/j.jand.2014.08.026, PMID: 25458748

[4] World Health Organization. Physical status: The use of and interpretation of anthropometry, report of a WHO expert committee. Geneva: World Health Organization (1995). 8594834

[5] Nguyen PH Menon P Ruel M. A situational review of infant and young child feeding practices and interventions in Viet Nam. Asia Pac J Clin Nutr. (2011) 20:359–74. PMID: 21859654

[6] ASEAN, UNICEF, WFP. ASEAN food and nutrition security report 2021 volume 2: Food and nutrition security country profiles. Jakarta: UNICEF (2022).

[7] Stewart CP Iannotti L Dewey KG Michaelsen KF Onyango AW. Contextualising complementary feeding in a broader framework for stunting prevention. Matern Child Nutr. (2013) 9:27–45. doi: 10.1111/mcn.12088, PMID: 24074316 PMC6860787

[8] Walson JL Berkley JA. The impact of malnutrition on childhood infections. Curr Opin Infect Dis. (2018) 31:231–6. doi: 10.1097/QCO.0000000000000448, PMID: 29570495 PMC6037284

[9] Dewey KG Begum K. Long-term consequences of stunting in early life. Matern Child Nutr. (2011) 7:5–18. doi: 10.1111/j.1740-8709.2011.00349.x, PMID: 21929633 PMC6860846

[10] Victora CG Adair L Fall C Hallal PC Martorell R Richter L. Maternal and child undernutrition: consequences for adult health and human capital. Lancet. (2008) 371:340–57. doi: 10.1016/S0140-6736(07)61692-4 18206223 PMC2258311

[11] Kristjansson E Francis DK Liberato S Jandu MB Welch V Batal M. Food supplementation for improving the physical and psychosocial health of socio-economically disadvantaged children aged three months to five years: a systematic review. Campbell Syst Rev. (2015) 11:1–226. doi: 10.4073/csr.2015.11 PMC6885042 25739460

[12] Keats EC Das JK Salam RA Lassi ZS Imdad A Black RE. Effective interventions to address maternal and child malnutrition: an update of the evidence. Lancet Child Adolesc Health. (2021) 5:367–84. doi: 10.1016/S2352-4642(20)30274-1, PMID: 33691083

[13] Ashworth A Ferguson E. Dietary counseling in the management of moderate malnourishment in children. Food Nutr Bull. (2009) 30:S405–33. doi: 10.1177/15648265090303S304, PMID: 19998865

[14] Lelijveld N Beedle A Farhikhtah A Elrayah EE Bourdaire J Aburto N. Systematic review of the treatment of moderate acute malnutrition using food products. Matern Child Nutr. (2020) 16:e12898. doi: 10.1111/mcn.12898, PMID: 31667981 PMC7038867

[15] Guldan GS Fan HC Ma X Ni ZZ Xiang X Tang MZ. Culturally appropriate nutrition education improves infant feeding and growth in rural Sichuan, China. J Nutr. (2000) 130:1204–11. doi: 10.1093/jn/130.5.1204, PMID: 10801920

[16] Santos I Victora CG Martines J Goncalves H Gigante DP Valle NJ. Nutrition counseling increases weight gain among Brazilian children. J Nutr. (2001) 131:2866–73. doi: 10.1093/jn/131.11.2866, PMID: 11694610

[17] Golden MH. Proposed recommended nutrient densities for moderately malnourished children. Food Nutr Bull. (2009) 30:S267–342. doi: 10.1177/15648265090303S302, PMID: 19998863

[18] World Health Organization. Energy and Protein Requirements: Report of a Joint FAO/WHO/UNU Expert Consultation. Geneva: World Health Organization (1985). 3937340

[19] van Vught AJAH Dagnelie PC Arts ICW Froberg K Andersen LB El-Naaman B. Dietary arginine and linear growth: the Copenhagen school child intervention study. Br J Nutr. (2013) 2013:1031–9. doi: 10.1017/S0007114512002942 23046689

[20] Schurgers LJ Teunissen KJ Hamulyák K Knapen MH Vik H Vermeer C. Vitamin K-containing dietary supplements: comparison of synthetic vitamin K1 and natto-derived menaquinone-7. Blood. (2007) 109:3279–83. doi: 10.1182/blood-2006-08-040709, PMID: 17158229

[21] Maresz K. Proper calcium use: vitamin K2 as a promoter of bone and cardiovascular health. Integr Med (Encinitas). (2015) 14:34–9. PMID: 26770129 PMC4566462

[22] Tsuchita H Suzuki T Kuwata T. The effect of casein phosphopeptides on calcium absorption from calcium-fortified milk in growing rats. Br J Nutr. (2001) 85:5–10. doi: 10.1079/BJN2000206, PMID: 11227028

[23] Cao Y Miao J Liu G Luo Z Xia Z Liu F. Bioactive peptides isolated from casein Phosphopeptides enhance calcium and magnesium uptake in Caco-2 cell monolayers. J Agric Food Chem. (2017) 65:2307–14. doi: 10.1021/acs.jafc.6b05711, PMID: 28218527

[24] Feng Y Zhang J Miao Y Guo W Feng G Yang Y. Prevention of zinc precipitation with calcium phosphate by casein hydrolysate improves zinc absorption in mouse small intestine ex vivo via a nanoparticle-mediated mechanism. J Agric Food Chem. (2020) 68:652–9. doi: 10.1021/acs.jafc.9b07097, PMID: 31869222

[25] Zhang Z Li F Hannon BA Hustead DS Aw MM Liu Z. Effect of Oral nutritional supplementation on growth in children with undernutrition: a systematic review and Meta-analysis. Nutrients. (2021) 13:3036. doi: 10.3390/nu13093036 34578914 PMC8468927

[26] Ip P Ho FKW Rao N Sun J Young ME Chow CB. Impact of nutritional supplements on cognitive development of children in developing countries: a meta-analysis. Sci Rep. (2017) 7:10611. doi: 10.1038/s41598-017-11023-4, PMID: 28878390 PMC5587553

[27] Ghosh AK Kishore B Shaikh I Satyavrat V Kumar A Shah T. Effect of oral nutritional supplementation on growth and recurrent upper respiratory tract infections in picky eating children at nutritional risk: a randomized, controlled trial. J Int Med Res. (2018) 46:2186–201. doi: 10.1177/0300060518757355, PMID: 29614897 PMC6023057

[28] Alarcon PA Lin L-H Noche M Jr Hernandez VC Cimafranca L Lam W. Effect of oral supplementation on catch-up growth in picky eaters. Clin Pediatr (Phila). (2003) 42:209–17. doi: 10.1177/000992280304200304, PMID: 12739919

[29] Khanna D Yalawar M Saibaba PV Bhatnagar S Ghosh A Jog P. Oral nutritional supplementation improves growth in children at malnutrition risk and with picky eating behaviors. Nutrients. (2021) 13:3590. doi: 10.3390/nu13103590, PMID: 34684591 PMC8538528

[30] Sheng X Tong M Zhao D Leung TF Zhang F Hays NP. Randomized controlled trial to compare growth parameters and nutrient adequacy in children with picky eating behaviors who received nutritional counseling with or without an oral nutritional supplement. Nutr Metab Insights. (2014) 7:85–94. doi: 10.4137/NMI.S15097, PMID: 25342910 PMC4196879

[31] Huynh DTT Estorninos E Capeding RZ Oliver JS Low YL Rosales FJ. Longitudinal growth and health outcomes in nutritionally at-risk children who received long-term nutritional intervention. J Hum Nutr Diet. (2015) 28:623–35. doi: 10.1111/jhn.12306, PMID: 25808062 PMC6680231

[32] Lebenthal Y Yackobovitch-Gavan M Lazar L Shalitin S Tenenbaum A Shamir R. Effect of a nutritional supplement on growth in short and lean prepubertal children: a prospective, randomized, double-blind, placebo-controlled study. J Pediatr. (2014) 165:1190–1193.e1. doi: 10.1016/j.jpeds.2014.08.011, PMID: 25241181

[33] Pham DT Hoang TN Ngo NT Nguyen LH Tran TQ Pham HM. Effect of oral nutritional supplementation on growth in Vietnamese children with stunting. Open Nutr J. (2019) 13:43–52. doi: 10.2174/1874288201913010043

[34] Mutunga M Rutishauser-Perera A Laillou A Prak S Berger J Wieringa FT. The relationship between wasting and stunting in Cambodian children: secondary analysis of longitudinal data of chil-dren below 24 months of age followed up until the age of 59 months. PLoS One. (2021) 16:e0259765. doi: 10.1371/journal.pone.0259765, PMID: 34794170 PMC8601787

[35] Huynh DT Estorninos E Capeding MR Oliver JS Low YL Rosales FJ. Impact of long-term use of oral nutritional supplement on nutritional adequacy, dietary diversity, food intake and growth of Filipino preschool children. J Nutritional Sci. (2016) 5:5. doi: 10.1017/jns.2016.6 PMC4891560 27293557

[36] Casale D Desmond C Richter LM. Catch-up growth in height and cognitive function: why definitions matter. Econ Hum Biol. (2020) 37:100853. doi: 10.1016/j.ehb.2020.100853, PMID: 32036257

[37] Cooke R Goulet O Huysentruyt K Joosten K Khadilkar AV Mao M. Catch-up growth in infants and young children with faltering growth: expert opinion to guide general clinicians. J Pediatr Gastroenterol Nutr. (2023) 77:7–15. doi: 10.1097/MPG.0000000000003784 36976274 PMC10259217

[38] Shepherd JA Wang L Fan B Gilsanz V Kalkwarf HJ Lappe J. Optimal monitoring time interval between DXA measures in children. J Bone Miner Res. (2011) 26:2745–52. doi: 10.1002/jbmr.473, PMID: 21773995 PMC3200454

[39] World Health Organization. WHO child growth standards: Length/height-for-age, weight-for-age, weight-for-length, weight-for-height and body mass index-for-age: Methods and development. Geneva: World Health Organization (2006).

[40] Schulz KF Grimes DA. Blinding in randomised trials: hiding who got what. Lancet. (2002) 359:696–700. doi: 10.1016/S0140-6736(02)07816-9, PMID: 11879884

[41] National Institute of Nutrition (Vietnam). 10 tips on proper nutrition for period 2013–2020 2013. Hanoi: Medical Publishing House (2020).

[42] National Institute of Nutrition (Vietnam). The Vietnamese food pyramid. Hanoi: Medical Publishing House (2010).

[43] National Institute of nutrition (Vietnam). Recommended dietary allowances for Vietnamese. Hanoi: Medical Publishing House (2016).

[44] World Health Organization. Preparation and use of food-based dietary guidelines: Report of a joint FAO/WHO consultation. Preparation and use of food-based dietary guidelines: Report of a joint FAO/WHO Consultation. Geneva: World Health Organization (1998). p. vi, 108–vi. 9795598

[45] Mehta NM Corkins MR Lyman B Malone A Goday PS Carney L. Defining pediatric malnutrition: a paradigm shift toward etiology-related definitions. J Parenter Enter Nutr. (2013) 37:460–81. doi: 10.1177/0148607113479972 23528324

[46] Leroy JL Ruel M Habicht JP Frongillo EA. Using height-for-age differences (HAD) instead of height-for-age z-scores (HAZ) for the meaningful measurement of population-level catch-up in linear growth in children less than 5 years of age. BMC Pediatr. (2015) 15:145. doi: 10.1186/s12887-015-0458-9, PMID: 26444012 PMC4595313

[47] Frongillo EA Leroy JL Lapping K. Appropriate use of linear growth measures to assess impact of interventions on child development and catch-up growth. Adv Nutr. (2019) 10:372–9. doi: 10.1093/advances/nmy093, PMID: 30805630 PMC6520037

[48] Gibson RS. Principles of nutritional assessment. USA: Oxford university press (2005).

[49] Frisancho AR. Anthropometric standards for the assessment of growth and nutritional status. United States of America: University of Michigan Press (1990).

[50] Bohannon RW Wang YC Bubela D Gershon RC. Handgrip strength: a population-based study of norms and age trajectories for 3- to 17-year-olds. Pediatr Phys Ther. (2017) 29:118–23. doi: 10.1097/PEP.0000000000000366, PMID: 28350764

[51] Shimizu S Kato-Nishimura K Mohri I Kagitani-Shimono K Tachibana M Ohno Y. Psychometric properties and population-based score distributions of the Japanese sleep questionnaire for preschoolers. Sleep Med. (2014) 15:451–8. doi: 10.1016/j.sleep.2013.05.020, PMID: 24636002

[52] LeBourgeois MK Harsh JR. Development and psychometric evaluation of the Children's sleep-wake scale. Sleep Health. (2016) 2:198–204. doi: 10.1016/j.sleh.2016.04.001, PMID: 28066802 PMC5215091

[53] Putnam SP Gartstein MA Rothbart MK. Measurement of fine-grained aspects of toddler temperament: the early childhood behavior questionnaire. Infant Behav Dev. (2006) 29:386–401. doi: 10.1016/j.infbeh.2006.01.004, PMID: 17138293 PMC4334385

[54] Rothbart MK Ahadi SA Hershey KL Fisher P. Investigations of temperament at three to seven years: the Children's behavior questionnaire. Child Dev. (2001) 72:1394–408. doi: 10.1111/1467-8624.00355 PMID: 11699677

[55] Moher D Schulz KF Altman D. The CONSORT statement: revised recommendations for improving the quality of reports of parallel-group randomized trials. JAMA. (2001) 285:1987–91. doi: 10.1001/jama.285.15.1987, PMID: 11308435

[56] Richard SA McCormick BJJ Murray-Kolb LE Bessong P Shrestha SK Mduma E. Influences on catch-up growth using relative versus absolute metrics: evidence from the MAL-ED cohort study. BMC Public Health. (2021) 21:1246. doi: 10.1186/s12889-021-11120-0, PMID: 34187407 PMC8240385

[57] Krause RJ Scott ME Sinisterra OT Koski KG. Preschool child growth attainment and velocity during an agriculture intervention in rural Panama may be diminished by soil-transmitted helminths. Front Public Health. (2023) 11:1122528. doi: 10.3389/fpubh.2023.1122528, PMID: 37829089 PMC10565504

[58] de Wit CC Sas TC Wit JM Cutfield WS. Patterns of catch-up growth. J Pediatr. (2013) 162:415–20. doi: 10.1016/j.jpeds.2012.10.014 23153864

[59] Samuel A Brouwer ID Feskens EJM Adish A Kebede A De-Regil LM. Effectiveness of a program intervention with reduced-Iron multiple micronutrient powders on Iron status, morbidity and growth in Young children in Ethiopia. Nutrients. (2018) 10:1508. doi: 10.3390/nu10101508, PMID: 30326609 PMC6212941

[60] Tessema M De Groote H Brouwer ID Feskens EJ Belayneh D Belachew T. Effect of quality protein maize on protein status and linear growth of Ethiopian children: A randomized controlled trial in linear growth failure of Ethopian children. Wageningen, the Netherlands: Wageningen University (2020).

[61] Leroy JL Frongillo EA Dewan P Black MM Waterland RA. Can children catch up from the consequences of undernourishment? Evidence from child linear growth, developmental epigenetics, and brain and neurocognitive development. Adv Nutr. (2020) 11:1032–41. doi: 10.1093/advances/nmaa020, PMID: 32584399 PMC7360439

[62] Isanaka S Hitchings MDT Berthé F Briend A Grais RF. Linear growth faltering and the role of weight attainment: prospective analysis of young children recovering from severe wasting in Niger. Matern Child Nutr. (2019) 15:e12817. doi: 10.1111/mcn.12817, PMID: 30903806 PMC6849732

[63] Thurstans S Sessions N Dolan C Sadler K Cichon B Isanaka S. The relationship between wasting and stunting in young children: a systematic review. Matern Child Nutr. (2022) 18:e13246. doi: 10.1111/mcn.13246, PMID: 34486229 PMC8710094

[64] Prado EL Dewey KG. Nutrition and brain development in early life. Nutr Rev. (2014) 72:267–84. doi: 10.1111/nure.12102, PMID: 24684384

[65] Pulakka A Cheung YB Maleta K Dewey KG Kumwenda C Bendabenda J. Effect of 12-month intervention with lipid-based nutrient supplement on the physical activity of Malawian toddlers: a randomised, controlled trial. Br J Nutr. (2017) 117:511–8. doi: 10.1017/S0007114517000290, PMID: 28382892 PMC5426340

[66] Jahari AB Saco-Pollitt C Husaini MA Pollitt E. Effects of an energy and micronutrient supplement on motor development and motor activity in undernourished children in Indonesia. Eur J Clin Nutr. (2000) 54:S60–8. doi: 10.1038/sj.ejcn.1601006, PMID: 10902988

[67] Aburto NJ Ramirez-Zea M Neufeld LM Flores-Ayala R. The effect of nutritional supplementation on physical activity and exploratory behavior of Mexican infants aged 8–12 months. Eur J Clin Nutr. (2010) 64:644–51. doi: 10.1038/ejcn.2010.52, PMID: 20354559

[68] Naila NN Mahfuz M Hossain M Arndt M Walson JL Nahar B. Improvement in appetite among stunted children receiving nutritional intervention in Bangladesh: results from a com-munity-based study. Eur J Clin Nutr. (2021) 75:1359–67. doi: 10.1038/s41430-020-00843-9, PMID: 34045689 PMC8416653

[69] Laillou A Pham TV Tran NT Le HT Wieringa F Rohner F. Micronutrient deficits are still public health issues among women and young children in Vietnam. PLoS One. (2012) 7:e34906. doi: 10.1371/journal.pone.0034906 22529954 PMC3328495

[70] Hoang NTD Orellana L Gibson RS Le TD Worsley A Sinclair AJ. Multiple micronutrient supplementation improves micronutrient status in primary school children in Hai Phong City, Vietnam: a randomised controlled trial. Sci Rep. (2021) 11:3728. doi: 10.1038/s41598-021-83129-9 33580103 PMC7881239

[71] Hermes FN Nunes EEM Melo CM. Sleep, nutritional status and eating behavior in children: a review study. Revista paulista de pediatria: orgao oficial da Sociedade de Pediatria de Sao Paulo. (2022) 40:e2020479. doi: 10.1590/1984-0462/2022/40/2020479 36102411 PMC9462407

[72] El Halal CDS Nunes ML. Sleep and weight-height development. J Pediatr. (2019) 95:2–9. doi: 10.1016/j.jped.2018.10.009 30528567

[73] Zhou Y Aris IM Tan SS Cai S Tint MT Krishnaswamy G. Sleep duration and growth outcomes across the first two years of life in the GUSTO study. Sleep Med. (2015) 16:1281–6. doi: 10.1016/j.sleep.2015.07.006, PMID: 26429758

[74] Lampl M Johnson ML. Infant growth in length follows prolonged sleep and increased naps. Sleep. (2011) 34:641–50. doi: 10.1093/sleep/34.5.641, PMID: 21532958 PMC3079944

[75] Jenni OG Molinari L Caflisch JA Largo RH. Sleep duration from ages 1 to 10 years: variability and stability in comparison with growth. Pediatrics. (2007) 120:e769–76. doi: 10.1542/peds.2006-3300, PMID: 17908734

[76] Kordas K Casavantes KM Mendoza C Lopez P Ronquillo D Rosado JL. The association between lead and micronutrient status, and children's sleep, classroom behavior, and activity. Arch Environ Occup Health. (2007) 62:105–12. doi: 10.3200/AEOH.62.2.105-112, PMID: 18316268

[77] Ji X Grandner MA Liu J. The relationship between micronutrient status and sleep patterns: a systematic review. Public Health Nutr. (2017) 20:687–701. doi: 10.1017/S1368980016002603, PMID: 27702409 PMC5675071

[78] Kordas K Siegel EH Olney DK Katz J Tielsch JM Kariger PK. The effects of iron and/or zinc supplementation on maternal reports of sleep in infants from Nepal and Zanzibar. J Dev Behav Pediatr. (2009) 30:131–9. doi: 10.1097/DBP.0b013e31819e6a48, PMID: 19322104 PMC2771202

[79] Al-Shawwa B Ehsan Z Ingram DG. Vitamin D and sleep in children. J Clin Sleep Med. (2020) 16:1119–23. doi: 10.5664/jcsm.8440, PMID: 32672533 PMC7954071

[80] Pourrostami K Heshmat R Derakhshanian H Ejtahed HS Shafiee G Safari O. The association between vitamin D status and sleep duration in school-aged children; the CASPIAN-V study. J Diabetes Metab Disord. (2023) 22:341–6. doi: 10.1007/s40200-022-01146-5, PMID: 37255800 PMC10225404

[81] Bravaccio C Terrone G Rizzo R Gulisano M Tosi M Curatolo P. Use of nutritional supplements based on melatonin, tryptophan and vitamin B6 (Melamil Tripto®) in children with primary chronic headache, with or without sleep disorders: a pilot study. Minerva Pediatr. (2020) 72:30–6. doi: 10.23736/S0026-4946.19.05533-6, PMID: 31621274

[82] Zhao H Lu C Yi C. Physical activity and sleep quality association in different populations: a meta-analysis. Int J Environ Res Public Health. (2023) 20:1864. doi: 10.3390/ijerph20031864, PMID: 36767229 PMC9914680

[83] Tham EKH Scheider N Broekman BFP. Infant sleep and its relation with cognition and growth: a narrative review. Nat Sci Sleep. (2017) 9:135–49. doi: 10.2147/NSS.S125992, PMID: 28553151 PMC5440010

[84] Suryawan A Jalaludin MY Poh BK Sanusi R Tan VMH Geurts JM. Malnutrition in early life and its neurodevelopmental and cognitive consequences: a scoping review. Nutr Res Rev. (2022) 35:136–49. doi: 10.1017/S0954422421000159, PMID: 34100353

[85] Larson LM Yousafzai AK. A meta-analysis of nutrition interventions on mental development of children under-two in low- and middle-income countries. Matern Child Nutr. (2017) 13:e12229. doi: 10.1111/mcn.12229, PMID: 26607403 PMC6866072

[86] Zhao Y He L Peng T Liu L Zhou H Xu Y. Nutritional status and function after high-calorie formula vs. Chinese food intervention in undernourished children with cerebral palsy. Front Nutr. (2022) 9:960763. doi: 10.3389/fnut.2022.960763, PMID: 36276835 PMC9582948

[87] Devaera Y Syaharutsa DM Jatmiko HK Sjarif DR. Comparing compliance and efficacy of isocaloric oral nutritional supplementation using 1.5 kcal/mL or 1 kcal/mL sip feeds in mildly to moderately malnourished Indonesian children: a randomized controlled trial. Pediatr Gastroenterol Hepatol Nutr. (2018) 21:315–20. doi: 10.5223/pghn.2018.21.4.315, PMID: 30345245 PMC6182471

[88] Vaivada T Lassi ZS Irfan O Salam RA Das JK Oh C. What can work and how? An overview of evidence-based interventions and delivery strategies to support health and human development from before conception to 20 years. Lancet. (2022) 399:1810–29. doi: 10.1016/S0140-6736(21)02725-2, PMID: 35489360

