# Supplementary material for: Oral nutritional supplementation with dietary counseling improves linear catch-up growth and health outcomes in children with or at risk of undernutrition: a randomized controlled trial
Source: Front Nutr. 2024 Jul 10;11:1341963. doi: 10.3389/fnut.2024.1341963 (PMC11266289; doi:10.3389/fnut.2024.1341963)
Supplement: Supplementary file 1 [file Table_1.pdf]

Supplementary Material

1 Supplementary Data

Table S1. Nutrient composition in two servings of oral nutrition supplement (ONS) and as a percentage of daily recommended nutrient intakes (RNI) for Vietnamese male children aged 3 – 5 years

**Nutrient**	**Amount in two servings (450 ml)**	**Percentage of RNI for Vietnamese males aged 3 – 5 years*, %**
Energy (kcal)	452	34.2
Protein (g)	13.48	53.9
Lipid (g)	17.62	40.5
Carbohydrate (g)	58.94	30.2
Calcium (mg)	450	75.0
Iron (mg)	6.3	114.5
Zinc (mg)	3.02	62.9
Vitamin A (mcg RE)	270	54.0
Vitamin D (mcg)	9.0	60.0
Vitamin E (IU)	10.4	231.1
Vitamin B1 (mg)	1.1	157.1
Vitamin B2 (mg)	0.96	120.0
Niacin (mg)	6.74	84.3
Pantothenic acid (mg)	3.14	104.7
Vitamin B6 (mg)	1.18	236.0
Folic acid (mcg)	108	122.4
Vitamin B12 (mcg)	1.36	136.0
Vitamin C (mg)	45.0	112.5

* Age- and sex-specific 2016 Vietnamese RNI

Table S2. Additional baseline sociodemographic and anthropometric characteristics of participants and households.

	**ONS + DC (n = 166)**	**DC only (n = 158)**
**Child characteristics**		
Ethnicity, n (%)		
Kinh	42 (26.6)	39 (23.6)
San Diu	50 (31.6)	49 (29.7)
Tay	30 (19.0)	28 (17.0)
Cao Lan	12 (7.6)	21 (12.7)
Other	24 (15.2)	28 (17.0)
Mode of delivery		
Vaginal	98 (62.0)	105 (63.6)
Caesarean section	60 (38.0)	60 (36.4)
Birth order	2.0 ± 0.1	2.0 ± 0.1
**Nutritional status**		
Stunting		
Normal	1 (0.6)	0 (0.0)
Mild	108 (66.7)	106 (64.6)
Moderate-Severe	53 (32.7)	58 (35.4)
Underweight		
Normal	0 (0.0)	1 (0.6)
Mild	111 (68.5)	111 (67.7)
Moderate-Severe	51 (31.5)	52 (31.7)
Wasting		
Normal	60 (37.0)	78 (47.6)
Mild	87 (53.7)	74 (45.1)
Moderate-Severe	15 (9.3)	12 (7.3)
**Parental characteristics**		
Mother's age (years)	30.8 ± 0.4	30.2 ± 0.4
Mother's height (cm)	154.47 ± 0.42	154.08 ± 0.36
Mother's weight (kg)	49.0 ± 0.4	48.3 ± 0.5
Mother's BMI (kg/m^2)	20.5 ± 0.2	20.3 ± 0.2
Father's age (years)	34.0 ± 0.4	33.5 ± 0.5
Father's height (cm)	164.15 ± 0.50	163.53 ± 0.42
Father's weight (kg)	59.1 ± 0.6	58.5 ± 0.6
Father's BMI (kg/m^2)	21.9 ± 0.2	21.9 ± 0.2
Father's highest level of education, n (%)		
Postgraduate Degree or Doctoral	0 (0.0)	0 (0.0)
College or University Degree	5 (3.2)	5 (3.1)
Associate or Technical Degree	10 (6.3)	5 (3.1)
High School Diploma	79 (50.0)	93 (57.1)
Secondary School	52 (32.9)	49 (30.1)
Primary School or less	9 (5.7)	8 (4.9)
Refused	3 (1.9)	3 (1.8)

Values are mean ± SE for continuous variables and n (%) for categorical variables. Sample sizes for some variables are less than the overall stated sample sizes. *p*-values in bold are p <0.05. BMI, body mass index; MUAC, mid-upper arm circumference; SE, standard error.

Table S3. Growth as assessed by various anthropometric indices at day 30 and 120^

	ONS + DC	DC Only	Difference or Odds Ratio (95% CI)	Change in ONS+DC/ DC only†	*p*-value
**WEIGHT INDICES**					
**Change at Day 30**					
**WAZ**	0.30 ± 0.02	0.18 ± 0.02	0.12 ± 0.03	1.67	**<0.001**
**WAP**	3.38 ± 0.27	1.84 ± 0.26	1.54 ± 0.36	1.84	**<0.001**
**WAD**	0.43 ± 0.03	0.22 ± 0.03	0.20 ± 0.05	1.95	**<0.001**
**Percentage with improved WAD**	83.5%	64.4%	2.92 (1.70, 5.01)	-	**<0.001**
**Percentage who improved to WAZ >-1**	15.7%	10.9%	4.86 (2.07, 11.39)*	-	**<0.001***
**Percentage who improved to WAZ >-2α**	36.0%	19.2%	N.S.	N.S.	0.529
**Change at Day 120**					
**WAZ**	0.30 ± 0.02	0.13 ± 0.02	0.17 ± 0.03	2.31	**<0.001**
**WAP**	3.55 ± 0.3	1.49 ± 0.29	2.06 ± 0.40	2.38	**<0.001**
**WAD**	0.32 ± 0.04	0.01 ± 0.04	0.31 ± 0.05	32.0	**<0.001**
**Percentage with improved WAD**	69.7%	44.4%	3.04 (1.89, 4.87)	-	**<0.001**
**Percentage who improved to WAZ >-1α**	18.1%	10.1%	4.86 (2.07, 11.39)*	-	**<0.001***
**Percentage who improved to WAZ >-2ß**	36.0%	19.2%	4.40 (1.40, 13.81)	-	**0.013**
**HEIGHT INDICES**					
**Change at Day 30**					
**HAZ**	0.20 ± 0.02	0.09 ± 0.02	0.11 ± 0.02	N.S.	0.876
**HAP**	0.75 ± 0.22	0.72 ± 0.21	0.03 ± 0.29	N.S.	0.929
**HAD**	0.12 ± 0.06	0.11 ± 0.06	0.01 ± 0.08	N.S.	0.843
**Percentage with improved HAD**	77.4%	59.3%	2.42 (1.46, 4.00)	-	0.001
**Percentage who improved to HAZ >-1α**	3.7%	6.7%	N.S.	-	0.427
**Percentage who improved to HAZ >-2ß**	16.3%	17.2%	N.S.	-	0.634
**Change at Day 120**					
**HAZ**	0.20 ± 0.02	0.09 ± 0.02	0.11 ± 0.02	2.22	**<0.001**
**HAP**	2.66 ± 0.26	1.11 ± 0.26	1.54 ± 0.35	2.40	**<0.001**
**HAD**	0.56 ± 0.07	0.10 ± 0.07	0.46 ± 0.09	5.60	**<0.001**
**Percentage with improved HAD**	77.4%	59.3%	2.42 (1.46, 4.00)	-	**0.001**
**Percentage who improved to HAZ >-1α**	16.2%	5.8%	8.06 (1.60, 40.62)	-	**0.021**
**Percentage who improved to HAZ >-2ß**	32.7%	19.0%	N.S.	-	0.185

^ Continuous data are presented as LSM ± SE from ANCOVA (site, treatment, gender, treatment*gender, age (in months), and respective baseline variable as covariates) models. Dichotomous data are presented as percentages with odds ratios of ONS+DC to DC-only group on a positive WAD/HAD change or improvement at day 30 or 120: GEE (site, treatment, gender, treatment*gender, visit, treatment*visit, with age (in months) and baseline value as covariates).
† LSM of change in the ONS + DC group divided by change in the DC only group. ONS + DC had twice the growth improvements in terms of percentiles and z-score increase from baseline in weight-for-age and height-for-age indices compared to DC only at day 120.
* Overall treatment effect p-value across day 30 and day 120 (treatment-by-visit interaction not significant) across Day 30 and 120. GEE, generalized estimating equation

[α] Sub-group analyses of z-score < - 1 and ≥ -2 at baseline.
[β] Sub-group analyses of z-score < -2 at baseline
p-values in bold are $p < 0.05$, underlined p-values are $p < 0.10$ and > 0.05.

ANCOVA, analysis of covariance; DC, dietary counseling; HAP, height-for-age percentile; HAZ, height-for-age z-score; LSM, least square mean; SE, standard error GEE, generalized estimating equation.

Table S4A. Subgroup analysis of HAZ, WAZ, WHZ ≥ - 2 at baseline (mild): recovery to normal nutritional status (HAZ, WAZ or WHZ ≥ - 1) at day 30 and 120

	**Numbers recovered to normal nutritional status, n / N (%)**			**Odds ratio of recovery to normal status (95% CI) †**	**p-value †**
	**ONS + DC**	**DC only**	**p-value ***		
**Mild Stunting at Baseline**					
Recovered to normal at day 30	4 / 107 (3.7)	7 / 105 (6.7)	0.337	-	0.427
Recovered to normal at day 120	17 / 105 (16.2)	6 / 104 (5.8)	**0.016**	8.06 (1.60, 40.62)	**0.021**
**Mild Underweight at Baseline**					
Recovered to normal at day 30	17 / 108 (15.7)	12 / 110 (10.9)	0.294	4.86 (2.07, 11.39)	**<0.001#**
Recovered to normal at day 120	19 / 105 (18.1)	11 / 109 (10.1)	<u>0.092</u>		
**Mild Wasting at Baseline**					
Recovered to normal at day 30	46 / 86 (53.5)	20 / 73 (27.4)	**< 0.001**	2.69 (1.62, 4.46)	**<0.001#**
Recovered to normal at day 120	29 / 84 (34.5)	14 / 72 (19.4)	**0.036**		

† Odds ratio of ONS + DC to control group recovering to a normal status (z-score ≥ -1) at day 30 or 120: GEE (site, treatment, gender, treatment*gender, visit, treatment*visit, with age (in months) and baseline value as covariates).
# Overall treatment effect p-value across day 30 and day 120 (treatment-by-visit interaction not significant) across Day 30 and 120. GEE, generalized estimating equation

Table S4B. Subgroup analysis with HAZ, WAZ, WHZ < - 2 at baseline (moderate-severe): reversal to mild undernutrition (HAZ, WAZ or WHZ ≥ - 1) at day 30 and 120

	**Numbers who reversed undernutrition (z-score ≥ -2) at day 30 or 120, n / N (%)**			**Odds ratio of reversal from undernutrition (95% CI) †**	**p-value †**
	**ONS + DC**	**DC only**	**p-value***		
**Stunting at Baseline**					
Reversed at day 30	8 / 49 (16.3)	10 / 58 (17.2)	0.8630	--	0.634
Reversed at day 120	16 / 49 (32.7)	11 / 58 (19.0)	0.1044	-	0.185
**Underweight at Baseline**					
Reversed at day 30	13 / 50 (26.0)	14 / 52 (26.9)	0.9159	-	0.529
Reserved at day 120	18 / 50 (36.0)	10 / 52 (19.2)	<u>0.0578</u>	4.40 (1.40, 13.81)	**0.013**
**Wasting at Baseline**					
Reversed at day 30	10 / 14 (71.4)	7 / 12 (58.3)	0.7063	-	0.234 #
Reversed at day 120	10 / 15 (66.7)	4 / 12 (33.3)	<u>0.0850</u>		

† Odds ratio of ONS + DC to control group having an improved status from baseline at day 30 or 120: GEE (site, treatment, gender, treatment*gender, visit, treatment*visit, with age (in months) and baseline value as covariates).
# Overall treatment effect p-value across day 30 and day 120 (treatment-by-visit interaction not significant) across Day 30 and 120. GEE, generalized estimating equation

Table S5. Parent-rated child's appetite, physical activity, energy levels, attentional focusing and sleep habits

	Follow-Up Visits	Intervention	Control	Difference	*p*-value
**Appetite, physical activity, and energy**					
Appetite	Baseline	6.14 ± 0.13	5.95 ± 0.13	0.19 ± 0.17	-
	Day 30/120	7.10 ± 0.09	6.50 ± 0.08	0.60 ± 0.12	**<0.001**
Physical activity	Baseline	7.79 ± 0.11	7.85 ± 0.11	-0.07 ± 0.14	-
	Day 30/120	8.21 ± 0.07	7.90 ± 0.06	0.31 ± 0.09	**<0.001**
Energy	Baseline	7.76 ± 0.12	7.82 ± 0.12	-0.06 ± 0.16	-
	Day 30/120	8.15 ± 0.07	7.94 ± 0.07	0.21 ± 0.09	**0.021**
**Attentional focusing**					
Mean scale score (≥24 to ≤36 months)	Baseline	4.09 ± 0.22	4.17 ± 0.24	-0.08 ± 0.29	-
	Day 30/120	4.56 ± 0.13	4.84 ± 0.14	-0.28 ± 0.17	0.120
Mean scale score (>36 to ≤60 months)	Baseline	4.41 ± 0.08	4.29 ± 0.08	0.11 ± 0.10	-
	Day 30/120	4.70 ± 0.05	4.55 ± 0.05	0.14 ± 0.07	**0.047**
**Sleep**					
Number night awakenings per night^	Baseline	0.5 ± 0.1	0.5 ± 0.1	-	-
	Day 30/120	0.2 ± 0.1	0.3 ± 0.1	Odds ratio: 1.62 (1.05 – 2.49)	**0.029**
Duration of night awakening (mins)	Baseline	2.29 ± 0.48	2.10 ± 0.47	0.20 ± 0.65	-
	Day 30/120	0.83 ± 0.27	1.99 ± 0.27	-1.16 ± 0.36	**0.001**
Total night sleep (hours)	Baseline	9.18 ± 0.06	9.22 ± 0.06	-0.04 ± 0.08	-
	Day 30/120	9.41 ± 0.05	9.26 ± 0.05	0.16 ± 0.06	**0.015**
Total day sleep (hours)	Baseline	1.87 ± 0.04	1.83 ± 0.04	0.04 ± 0.05	-
	Day 30/120	1.85 ± 0.05	1.83 ± 0.04	0.03 ± 0.06	0.672
Overall sleep quality (10-point VAS)	Baseline	7.97 ± 0.11	8.17 ± 0.10	-0.20 ± 0.14	-
	Day 30/120	8.81 ± 0.06	8.39 ± 0.06	0.41 ± 0.08	**<0.001**

LSM ± SE. Unless otherwise stated. Day 30/120 analyses: repeated-measures ANCOVA (site, treatment, gender, treatment*gender, visit, treatment*visit and with age (in months) and respective baseline variable as covariates). *p*-values in bold are $p < 0.05$.
^Observed mean ± SE for baseline and day 120 values. Baseline analysis: chi-square test; and day 30/120 analysis: GEE (site, treatment, gender, treatment*gender, visit, treatment*visit, with age (in months) and baseline value as covariates).
ANCOVA, analysis of covariance; GEE, generalized estimating equation; LSM, least squares mean; SE, standard error.

Table S6. Parental satisfaction with child's health and growth at baseline and at post-baseline visits

	Visit	ONS + DC	DC Only	Difference	*p*-value
Satisfied with child's weight	Baseline	5.92 ± 0.12	5.91 ± 0.12	0.01 ± 0.16	-
	Day 30/120	6.97 ± 0.09	6.53 ± 0.09	0.44 ± 0.12	**<0.001**
Satisfied with child's height	Baseline	6.05 ± 0.13	6.02 ± 0.12	0.02 ± 0.17	-
	Day 30/120	7.13 ± 0.09	6.74 ± 0.08	0.39 ± 0.11	**<0.00**1
Satisfied with child's growth	Baseline	6.34 ± 0.12	6.30 ± 0.12	0.04 ± 0.16	-
	Day 30/120	7.23 ± 0.09	6.75 ± 0.08	0.49 ± 0.12	**<0.001**
Satisfied with child's muscle health	Baseline	6.28 ± 0.12	6.15 ± 0.12	0.14 ± 0.12	-
	Day 30/120	7.20 ± 0.10	6.80 ± 0.10	0.36 ± 0.11	**0.001**
Satisfied with child's bone health	Baseline	6.83 ± 0.12	6.80 ± 0.12	0.03 ± 0.16	-
	Day 30/120	7.50 ± 0.10	7.10 ± 0.10	0.39 ± 0.10	**<0.001**

Data are presented as LSM ± SE. Day 30/120 analyses: repeated-measures ANCOVA (site, treatment, gender, treatment*gender, visit, treatment*visit and with age (in months) and respective baseline variable as covariates). p-values in bold are $p<0.05$.
DC, dietary counseling; LSM, least squares mean; ONS, oral nutrition supplement; SE, standard error.

Table S7. Parental evaluation of child's health and growth since intervention from baseline

	ONS + DC	DC Only	Difference	*p*-value
**Parental evaluation of child's health and growth since the intervention**				
My child achieved good growth	7.80 ± 0.10	6.90 ± 0.10	0.86 ± 0.12	**<0.001**
My child has firmer muscle	7.70 ± 0.10	7.00 ± 0.10	0.71 ± 0.11	**<0.001**
My child has stronger bones	7.80 ± 0.10	7.10 ± 0.10	0.66 ± 0.11	**<0.001**
My child has stronger teeth	7.10 ± 0.10	6.60 ± 0.10	0.53 ± 0.15	**<0.001**
My child is more alert/curious/eager	8.10 ± 0.10	7.70 ± 0.10	0.41 ± 0.10	**<0.001**
My child has healthier looking skin	7.70 ± 0.10	7.20 ± 0.10	0.55 ± 0.10	**<0.001**
My child has healthier looking nails	7.60 ± 0.10	7.10 ± 0.10	0.47 ± 0.10	**<0.001**
My child has healthier looking hair	7.70 ± 0.10	7.20 ± 0.10	0.54 ± 0.10	**<0.001**
My child has healthier looking eyes	7.90 ± 0.10	7.50 ± 0.10	0.37 ± 0.10	**<0.001**

Data are presented as LSM ± SE. Repeated-measures ANCOVA (site, treatment, gender, treatment*gender, visit, treatment*visit and with age (in months)) *p*-values in bold are p <0.05.
DC, dietary counseling; LSM, least squares mean; ONS, oral nutrition supplement; SE, standard error.
